# Gut microbial metabolism of bile acids modifies the effect of Mediterranean diet interventions on cardiometabolic risk in a randomized controlled trial

**DOI:** 10.1080/19490976.2024.2426610

**Published:** 2024-11-13

**Authors:** Peipei Gao, Ehud Rinott, Danyue Dong, Zhendong Mei, Fenglei Wang, Yuxi Liu, Omer Kamer, Anat Yaskolka Meir, Kieran M. Tuohy, Matthias Blüher, Michael Stumvoll, Meir J. Stampfer, Iris Shai, Dong D. Wang

**Affiliations:** a Department of Nutrition, Harvard T.H. Chan School of Public Health, Boston, MA, USA; b Human Phenome Institute, Fudan University, Shanghai, China; c Faculty of Health Sciences, The Health & Nutrition Innovative International Research Center, Ben-Gurion University of the Negev, Be’er Sheva, Israel; d Channing Division of Network Medicine, Department of Medicine, Brigham and Women’s Hospital and Harvard Medical School, Boston, MA, USA; e Broad Institute of MIT and Harvard, Cambridge, MA, USA; f Department of Epidemiology, Harvard T.H. Chan School of Public Health, Boston, MA, USA; g School of Food Science and Nutrition, University of Leeds, Leeds, UK; h Helmholtz Institute for Metabolic, Obesity and Vascular Research (HI-MAG), Helmholtz Zentrum München, University of Leipzig and University Hospital Leipzig, Leipzig, Germany; i Medical Department III-Endocrinology, Nephrology, Rheumatology, University of Leipzig Medical Center, Leipzig, Germany

**Keywords:** Bile acids, Mediterranean diet, gut microbiome, cardiometabolic health

## Abstract

Bile acids (BAs) undergo extensive microbial metabolism in the gut and exert hormone-like functions on physiological processes underlying metabolic risk. However, the extent to which gut BA profiles predict cardiometabolic risk and explain individual responses to dietary interventions in humans is still unclear. In the DIRECT-PLUS Trial, we conducted a multi-omics analysis of 284 participants randomized into three groups: healthy dietary guidelines and two Mediterranean diet (MedDiet) groups. We longitudinally measured 44 fecal BAs using liquid chromatography-mass spectrometry, the gut microbiome through shotgun metagenomic sequencing, and body adiposity and serum lipids at baseline, 6, and 18 months. Fecal levels of 14 BAs, such as lithocholic acid and ursodeoxycholic acid, were prospectively associated with body mass index (BMI) and serum lipid profiles (false discovery rate [*q*]<0.05). Baseline fecal BA levels significantly modified the beneficial effects of the MedDiet; for example, BMI reduction induced by MedDiet interventions was more pronounced in individuals with lower 12-dehydrocholic acid levels (*q*-interaction <0.001). We confirmed that the gut microbiome is a major modifier of the secondary BA pool in humans. Furthermore, the association of fecal BAs with body adiposity and serum lipids varied significantly in individuals with different abundances of gut microbes carrying BA metabolism enzymes, e.g. several *Ruminococcus* spp. In summary, our study identifies novel predictive biomarkers for cardiometabolic risk and offers new mechanistic insights to guide personalized dietary interventions.

## Introduction

Bile acids (BAs) are initially synthesized from cholesterol in the liver, largely in response to dietary intake.^1^ The primary bile acids (PBAs) are then released into the gastrointestinal tract, where microbial enzymes catalyze modifications such as deconjugation and dehydroxylation, resulting in secondary bile acids (SBAs).^2^ Additional microbial modifications, including oxidation and epimerization by bacterial hydroxysteroid dehydrogenases (HSDH), enhance the diversity and composition of the BA pool.^3^ A significant portion of these SBAs is reabsorbed into the peripheral circulation and returned to the liver.^2,3^ Both PBAs and SBAs play pivotal roles in modulating host lipid, glucose, and energy homeostasis,^4,5^ through the activation of nuclear receptors – farnesoid X receptor (FXR), pregnane X receptor, and vitamin D receptor – and a G protein-coupled receptor (TGR5),^3,4^ as well as through the regulation of glucagon-like peptide (GLP-1) synthesis.^6,7^ Moreover, BAs maintain a bidirectional interaction with the gut microbiome, wherein the microbial composition and functionality influence the dynamics of the BA pool, and conversely, BAs modify the microbial community structure.^8,9^ However, most current evidence for the interactions and their implication in cardiometabolic health is from *in vivo* studies. Prospective studies on the associations among the BA pool in the gut, the microbial community, and cardiometabolic health in humans are scarce. Furthermore, given the potential role of BAs in modulating susceptibility to cardiometabolic diseases and their close interaction with dietary intake,^9,10^ there is a significant interest in examining whether and to what extent baseline BA levels can explain inter-individual differences in dietary response. This question is central to precision nutrition and would be ideally addressed in a randomized controlled trial (RCT) setting. Yet, to date, no RCT has specifically examined this research question.

Here, we present an analysis of fecal BAs, the gut microbiome, body adiposity, and lipid metabolism biomarkers measured longitudinally in 284 participants from the DIRECT-PLUS Trial. Participants were randomized into three groups: one following healthy dietary guidelines and two following variations of Mediterranean diet (MedDiet). Our prior publications have reported that MedDiet, compared to HDG, led to significant reductions in body adiposity and favorable changes in serum cardiometabolic biomarkers over 18 months.^11^ In this study, our initial findings indicate that baseline levels of 14 fecal BAs were prospectively associated with body adiposity and serum biomarkers of lipid metabolism. Furthermore, the study revealed that the beneficial effects of MedDiet interventions on cardiometabolic health outcomes varied according to the baseline levels of BAs. Additionally, we confirmed that the gut microbiome significantly modifies the secondary BA pool in humans and that the associations between fecal BAs, body adiposity, and serum lipids differ depending on the abundance of gut microbes involved in BA metabolism. Adopting a multi-omics approach, our study provides a comprehensive investigation of gut microbial metabolism of BAs and its implications for predicting cardiometabolic risk and guiding personalized nutrition interventions within a rigorously conducted RCT in humans.

## Results

### Characterization of fecal bile acids, the gut microbiome profiles, and cardiometabolic biomarkers in a Mediterranean diet intervention trial

**Figure 1.**
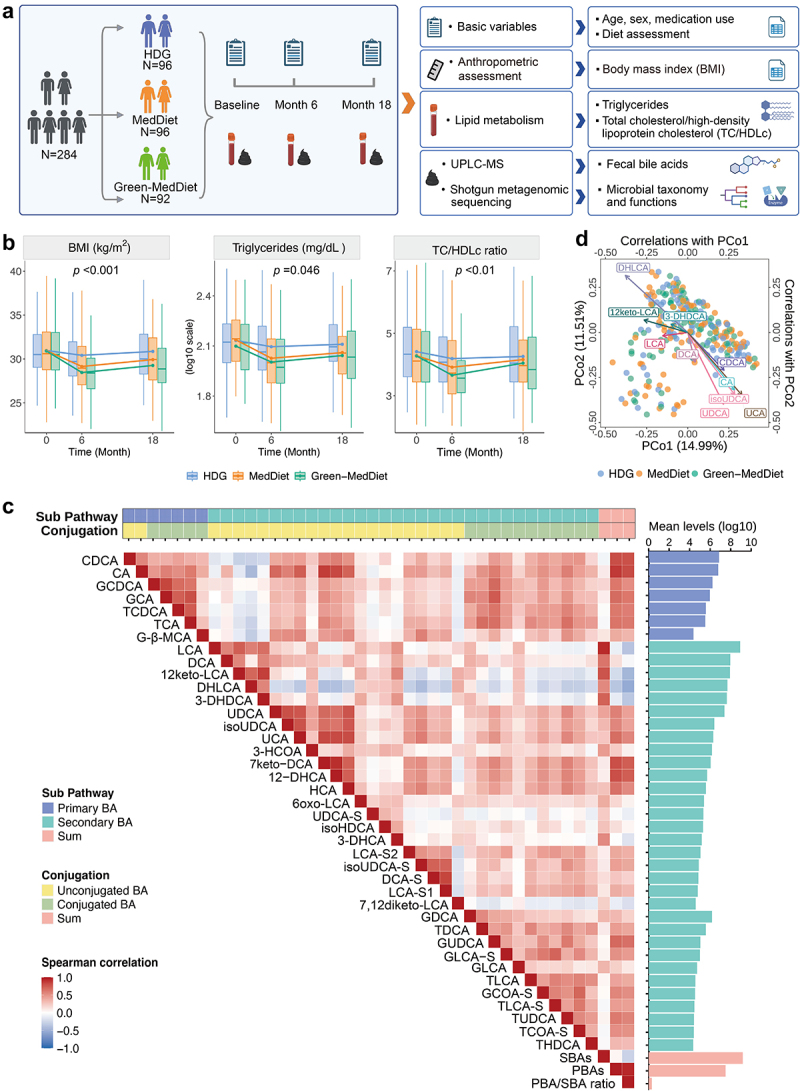
Study design for linking Mediterranean diet, gut microbial metabolism of bile acids, and cardiometabolic health in the DIRECT-PLUS trial. (a) Overview of our study that incorporates randomized dietary interventions and longitudinal measurements of the gut microbiome, fecal bile acids (BAs), and cardiometabolic biomarkers. We profiled fecal BAs using ultra-high performance liquid chromatography-mass spectrometry (UPLC-MS) and microbial taxonomy and functions using shotgun metagenomic sequencing. This study measured body adiposity and serum lipid biomarkers. (b) Temporal trends in body adiposity and lipid biomarkers in dietary intervention groups (*N* = 294). The *p*-values for the interaction terms between intervention groups and time were derived from the Wald test in the multivariable-adjusted generalized estimating equation models. The center of each box plot was the median value, with the boxes displaying the interquartile ranges (IQRs), upper and lower whiskers indicating 1.5 times the IQR from above the upper quartile and below the lower quartile, respectively. (c) Spearman correlations among fecal levels of 39 individual BAs, primary bile acids (PBAs), secondary bile acids (SBAs), and PBA/SBA ratio, with red color indicating positive correlations and blue color indicating inverse correlations. The bar plots on the right side display the mean levels for each BA. (d) Correlations between the overall gut microbiome configuration and the top ten most abundant fecal BAs. The structure of the gut microbiome, as illustrated by the principal coordinate analysis (PCoA) plot, is colored in correspondence to dietary intervention arms. The arrow endpoints represent Spearman correlation coefficients between each BA and the 1^st^ and 2^nd^ principal coordinate (PCo1 and PCo2) values. Among these ten BAs, nine showed significant correlations with either the PCo1 or PCo2 (*q* < 0.05). The PCoA was conducted based on the Bray-Curtis dissimilarity calculated from species-level microbial features. Abbreviations: BMI, body mass index; CA, cholic acid; CDCA, chenodeoxycholic acid; HDG, healthy dietary guidelines; MedDiet, Mediterranean diet; PBAs, primary bile acids; SBAs, secondary bile acids; *q*, false discovery rate adjusted *p*-value; TC/HDLc, ratio of total cholesterol to high-density lipoprotein cholesterol; UPLC-MS, ultrahigh performance liquid chromatography with tandem mass spectrometry; the original names of other BAs are detailed in Table S3.

To investigate the interplay between gut microbial metabolism of BAs, dietary interventions, and cardiometabolic risk, we assembled longitudinally collected data on fecal BA levels, microbial profiles, body adiposity, and serum biomarkers of lipid metabolism in 284 participants from the DIRECT-PLUS Trial (Figure 1a and Table S1). The DIRECT-PLUS trial (clinicaltrials.gov ID: NCT03020186) was designed to evaluate the effects of MedDiet versus standard healthy dietary guidelines (HDG, control group) on body adiposity and biomarkers of cardiometabolic risk (Figure 1b and Table S2; Methods). The study population was 88% men, reflecting the nature of the workplace, with an average age of 51.2 years (range 31.9 to 82.0 years) and a mean BMI of 31.2 kg/m^2^ (SD = 3.9) at baseline. The baseline characteristics, including age, sex, medication use, physical activity and dietary fat intake, were comparable across intervention group assignments.

We included 39 fecal BAs (from a total of 44 BAs measured) detected above the detection threshold in more than 20% of all participants (Table S3), comprising 7 PBAs and 32 SBAs (Figure 1c, Fig. S1 and Table S3). The PBAs included two major human BAs of cholic acid (CA) and chenodeoxycholic acid (CDCA), along with five other conjugated BA forms; the SBAs consisted of 11 conjugated and 21 unconjugated forms. Their Spearman correlation coefficients ranged from −0.52 to 0.94, with the majority exhibiting positive correlations (82.0%, Figure 1c and Table S4). Fecal levels of lithocholic acid (LCA), which were the highest on average (Figure 1c, Table S4–5 and Fig. S2A), showed a notably high correlation of 0.93 with the sum of SBAs. Furthermore, transformations among BAs have been demonstrated by *in vitro* and *in vivo* studies,^3,12^ such as 7-keto-deoxycholic acid (7keto-DCA) being enzymatically converted from CA by 7α-HSDH and further metabolized into ursocholic acid (UCA) by 7β-HSDH. Correspondingly, our analysis identified strong correlations in fecal levels between CA and its derivative 7keto-DCA (*q* < 0.001), as well as between 7keto-DCA and UCA (*q* < 0.001).

We measured the gut microbiome through shotgun metagenomic sequencing of stool samples. After quality control, the microbial profiling workflow yielded 151 microbial species, 1570 level-4 Enzyme Commission categories (ECs), and 372 biochemical pathways (Methods). As expected, the ten most abundant species together accounted for an average of 46.7% of the overall microbial community abundance (Fig. S2B and Table S5). The overall structure of the gut microbiome was correlated to fecal BA levels, and among the top ten most abundant fecal BAs, nine BAs showed significant correlations with the gut microbiome configuration (Figure 1d, *q* < 0.05).

### Baseline levels of fecal bile acids were prospectively associated with body adiposity and lipid biomarkers

We first investigated whether baseline BA levels measured from stool samples were prospectively associated with body adiposity and lipid biomarkers during follow-up. Although previous population-based and clinical studies have linked circulating BAs to obesity and cardiometabolic risk,^13–15^ it remains largely unknown whether fecal BA levels, as measures of gut microbial metabolism of BAs, can predict individuals’ cardiometabolic disease risk. To address this, we constructed generalized estimating equation (GEE) models to account for the longitudinal design of our study.

**Figure 2.**
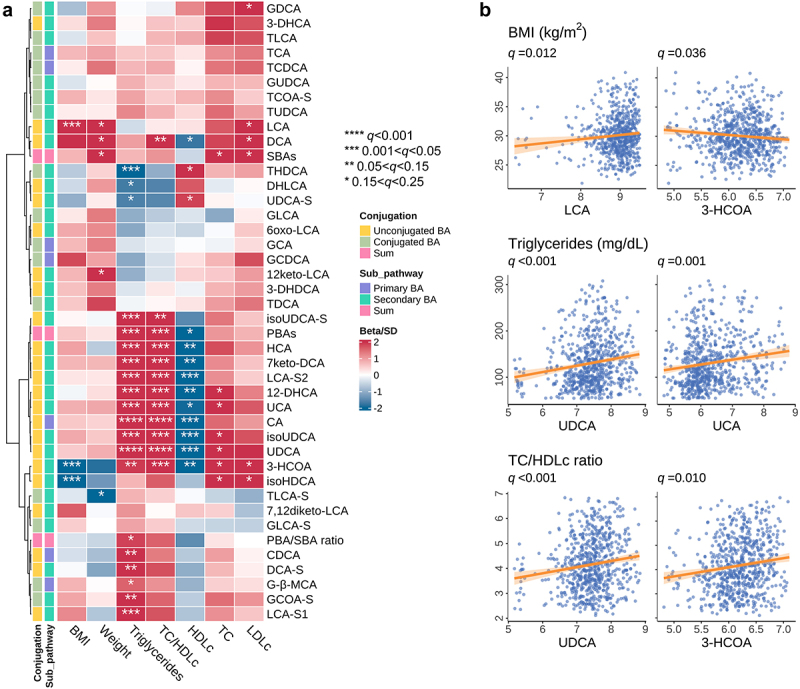
Fecal bile acid levels were prospectively associated with body adiposity and lipid profiles during the 18-month follow-up in the DIRECT-PLUS trial. (a) Prospective associations between fecal bile acid (BA) levels measured at baseline and longitudinally measured body adiposity, including body mass index (BMI) and body weight, as well as lipid biomarkers, including triglycerides, total cholesterol (TC), high-density lipoprotein cholesterol (HDLc), TC/HDLc ratio, and low-density lipoprotein cholesterol (LDLc). Q-values (false discovery rate adjusted *p*-value) were derived from generalized estimating equation models, which included age, sex, Mediterranean diet index, time, antibiotic use, metformin use, and lipid-lowering medication use as covariates, with participant identifiers accounting for within-subject correlations. (b) Select associations between fecal BA levels (log_10_ scale) measured at baseline and body adiposity (BMI) and lipid biomarkers (triglycerides and TC/HDLc ratio). Abbreviations: LCA, lithocholic acid; 3-HCOA, 3beta-hydroxy-5-cholenoic acid; UDCA, ursodeoxycholic acid; UCA, ursocholic acid.

Among all the 39 BAs, 14 were prospectively associated with BMI (including three SBAs) and serum lipid biomarkers (including one PBA and 11 SBAs) after multiple-comparison adjustment (*q* < 0.05, Figure 2a-b and Table S6). We observed significant associations between levels of LCA and two hyocholic acid (HCA) isoforms (3beta-hydroxy-5-cholenoic acid [3-HCOA], and isohyodeoxycholic acid [isoHDCA]) with BMI (*q* < 0.05). For example, participants in the highest tertile of LCA level, compared to those in the lowest tertile, had a 2.3 kg/m^2^ higher BMI (*q* = 0.009, Table S7). In addition, we identified specific BA species within the ursodeoxycholic acid (UDCA; including UDCA, isoUDCA, and isoursodeoxycholic acid-sulfate [isoUDCA-S]), HCA (including HCA, and 3-HCOA), and LCA (including lithocholic sulfate 1 [LCA-S1], or lithocholic sulfate 2 [LCA-S2]) families that were positively associated with triglycerides or TC/HDLc (*q* < 0.05). For instance, individuals in the highest versus lowest tertiles of UDCA level had 38.8 mg/dL higher triglycerides (*q* = 0.001) and a 0.6 higher TC/HDLc ratio (*q* = 0.001). It is noteworthy that LCA, UDCA, and HCA species have been reported by animal or *in vitro* models to activate with TGR5 and FXR signaling to regulate metabolic homeostasis.^6,16–18^ Additionally, the association between fecal BAs and TC/HDLc appeared to be mainly driven by HDLc levels. Furthermore, results from models that further adjusted for physical activity (Fig. S3A), dietary fat intake (Fig. S3B), or the alanine aminotransferase/aspartate aminotransferase (ALT/AST) ratio (Fig. S3C) were highly correlated (all coefficients > 0.83) between the beta coefficients from the primary analysis and those from three sensitivity analyses. In another sensitivity analysis, we removed samples with missing values for BAs that had a missing rate between 10% and 80%. The results showed that the prospective associations between these fecal BAs and body adiposity, as well as lipid biomarkers, were largely consistent with those from the primary analysis (Fig. S3D).

### The effects of MedDiet interventions on body adiposity and lipid metabolism vary among individuals with different baseline fecal bile acid levels

In the DIRECT-PLUS Trial, we previously reported that MedDiet, compared to HDG, led to significant favorable changes in BMI and serum lipid biomarkers (Figure 1b).^19,20^ However, we observed substantial variability in individual responses to the dietary interventions (Fig. S4). Given that both PBAs and gut microbial metabolism of BAs have intricate interrelationships with a healthy diet, an individual’s preexisting gut microbial capacity to metabolize BAs could modify the effects of the MedDiet.^3,8,21^ Therefore, our subsequent analysis sought to determine the extent to which baseline levels of fecal BAs could explain the differences in individuals’ responses, in terms of changes in BMI and lipid profiles, to the MedDiet interventions.

**Figure 3.**
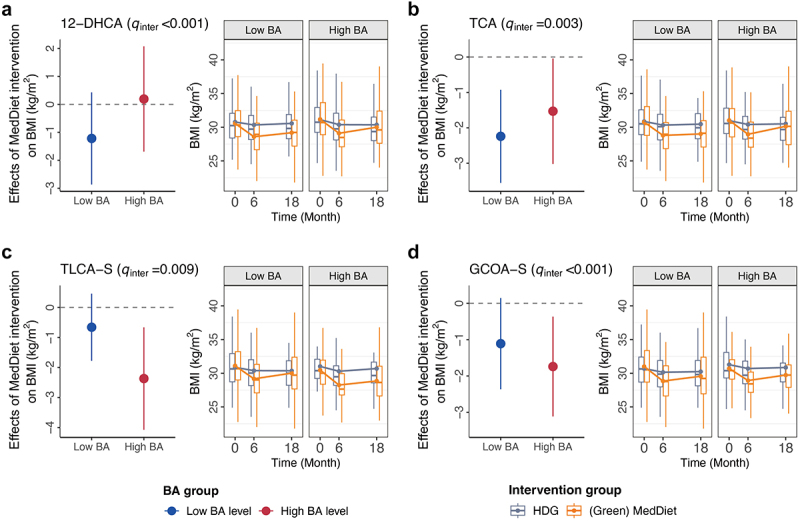
The effects of Mediterranean diet interventions on body adiposity and lipid metabolism vary among individuals with different baseline fecal bile acid levels. The dots in the plot represent the beta coefficients for the effect of the Mediterranean diet (MedDiet) intervention on body mass index (BMI) from multivariable-adjusted generalized estimating equation (GEE) models, with error bars indicating the upper and lower limits of the 95% confidence intervals (CIs). The box plots show the temporal trends in BMI in two Mediterranean diet groups combined compared to the healthy dietary guidelines group stratified by BA levels. Low vs. high BA levels were determined based on the median values of each BA. The center of each box plot was the median value, with the boxes displaying the interquartile ranges (IQR), upper and lower whiskers indicating 1.5 times the IQR from above the upper quartile and below the lower quartile, respectively. We calculated the *p*-values for the interaction terms between intervention group assignment and time using the multivariable-adjusted generalized estimating equation models and then performed multiple-comparison adjustment using the Benjamini-Hochberg procedure to calculate false discovery rate adjusted *p* values (*q*_inter_). Abbreviations: GCOA-S, glycocholenoic acid sulfate; HDG, healthy dietary guidelines; MedDiet, Mediterranean diet; TCA, taurocholic acid; TLCA-S, taurolithocholic acid 3-sulfate; 12-DHCA, 12-dehydrocholic acid.

We employed a statistical framework commonly used in population-based studies^22^ to test for interaction and examine whether the effects of MedDiet, compared to HDG, on body adiposity or lipid biomarkers vary among individuals with different baseline levels. We found that the effects of MedDiet in improving BMI were significantly more pronounced in participants with lower levels of 12-dehydrocholic acid (12-DHCA, *q* for interaction < 0.001, Figure 3 and Table S8) and taurocholic acid (TCA, *q* for interaction = 0.003). In comparison, more pronounced effects were observed in those with higher levels of sulfation isoforms, including taurolithocholic acid 3-sulfate (TLCA-S, *q* for interaction = 0.009) and glycocholenoic-sulfate (GCOA-S, *q* for interaction < 0.001). Moreover, the baseline fecal levels of various BAs were found to modulate MedDiet’s effects on lipid profiles (Fig. S5 and Table S8). The effect of MedDiet for lowering triglycerides varied depending on the levels of 12-DHCA and LCA (*p* for interaction < 0.05). Significant interaction patterns were also observed among BAs with other lipid biomarkers. For example, the effect for lowering TC/HDLc was stratified by TCA and taurochenodeoxycholic acid (TCDCA) groups, the effect for lowering TC varied by GCOA-S and TCDCA levels, and the effect on LDLc varied by GCOA-S and dehydrolithocholic acid (DHLCA) groups. Subgroup analyses supported the potential interactions between baseline BAs and MedDiet interventions on body adiposity and lipid biomarkers. The effects observed in the main analysis were consistent across subgroups stratified by baseline levels of MedDiet adherence index, BMI, triglycerides, and TC/HDL-C ratio, as well as in different age groups, and those in male participants only (as 88.4% of participants were male), with results showing similar directional effects (Fig. S5-S6). Another sensitivity analysis examined the effects of dietary intervention on BMI by baseline levels of BAs for MedDiet and Green-MedDiet separately. We found that the effects of the dietary interventions varied in the same direction and with similar magnitude for both MedDiet and Green-MedDiet (Fig. S7). Our study identified diverse groups of BAs that can explain inter-individual variations in response to dietary interventions, highlighting their roles as new response biomarkers and offering new mechanistic insights for personalized dietary intervention.

### Mediterranean and healthy dietary guidelines diets differentially alter fecal bile acid levels

Next, we sought to understand if different dietary patterns had distinct effects on the temporal changes in fecal BAs. We used GEE models with BA levels as the dependent variable and included the same covariables described previously, with participant identifiers to deal with within-subject correlations. The models specifically incorporated an interaction term between dietary intervention group assignment and the time variable (representing the slope of changes in BA levels over time). A significant effect estimate of the interaction term indicated that the different dietary patterns altered fecal BA levels differentially.

Notably, we observed a reduction in BA levels six months after the intervention, followed by an increase from month 6 to month 18 (Fig. S8 and Table S3). This trend mirrors the temporal patterns in BMI and serum lipid biomarkers induced by dietary interventions. During the 18-month follow-up, the MedDiet interventions, compared to HDG, led to a more substantial decrease in the levels of CA, 7keto-DCA, and 12-DHCA (*q*-interaction between intervention assignment and time < 0.05, Fig. S9). In addition, we found suggestive evidence that MedDiet interventions led to more pronounced effects in modulating fecal levels of SBA, including LCA, UCA, UDCA, isoUDCA, 7keto-DCA, and 12-DHCA (Fig. S8 and Table S3, *p* < 0.05), which may be attributed to the higher content of prebiotic nutrients, such as flavonoids and pectin, in the MedDiet.^23^

### The gut microbiome modulates the secondary bile acid pool and its association with cardiometabolic risk

**Figure 4.**
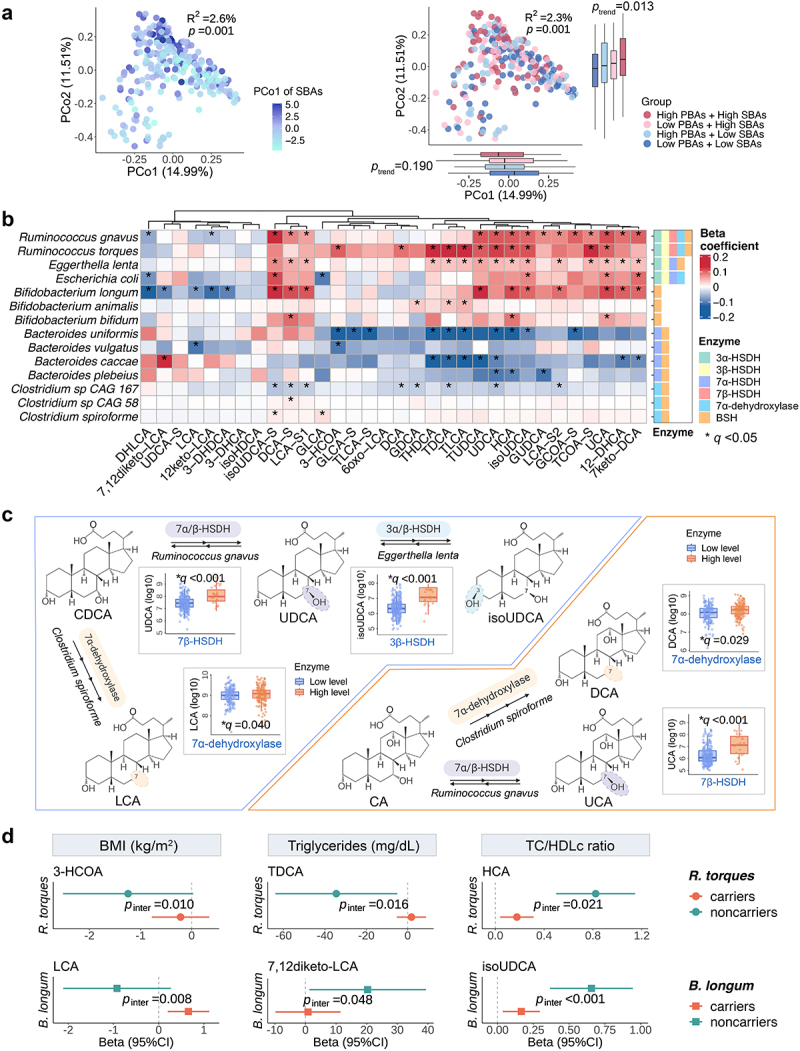
Interrelationships between the gut microbiome, fecal bile acids, body adiposity and serum lipid profiles. (a) The association between overall gut microbiome configuration and bile acids (BAs). Principal coordinate analysis (PCoA) scatter plots, generated based on Bray-Curtis dissimilarity calculated from species-level microbial features, are colored by the level of the major variation of secondary BAs (SBAs, left plot) and by BAs categories (right plot). The major variation of SBAs was quantified by the 1st principal coordinate (PCo1, explained variance: 29.44%) score from PCoAs based on the Euclidean distance of SBAs. Based on the median values for the sum of primary bile acids (PBAs) and SBAs, BA levels were categorized into four categories. The significance level for the association of SBAs with the gut microbiota was assessed using permutational multivariate analysis of variance (PERMANOVA) with 999 permutations. The *p*-trend value was derived from the Wald test in the multivariable-adjusted linear regression model. (b) Associations between individual microbial species involved in SBA metabolism and fecal SBA levels. The right-hand stacked barplot displays the BA metabolism enzymes encoded by each species. We calculated the *p*-values from the multivariable-adjusted linear models and then performed multiple-comparison adjustment using the Benjamini-Hochberg procedure to calculate false discovery rate (FDR) adjusted *p* values (*q* values) (c) Associations between the BA metabolism enzymes and fecal BA levels as illustrated by simplified biosynthetic pathways that involve hydroxysteroid dehydrogenase (HSDH) and 7α-dehydroxylase and major SBA-processing bacteria. The box plots with dots show the fecal BA levels grouped by high or low enzyme levels that are determined by the median value of the relative abundance of each enzyme. The *q*-values were derived from multivariable-adjusted linear regression models. Each box plot centers on the median value, with the boxes displaying the interquartile ranges (IQRs), upper and lower whiskers indicating 1.5 times the IQR from above the upper quartile and below the lower quartile, respectively. (d) Selected SBA-processing bacteria modify the association of fecal SBAs with body mass index (BMI) and serum lipid biomarkers. The dot plots with whiskers indicate the difference in BMI per one-standard deviation increment in BA levels with 95% confidence intervals in carriers and noncarriers of the species. Abbreviations: BAs, bile acids; BMI, body mass index; CA, cholic acid; CDCA, chenodeoxycholic acid; DCA, deoxycholic acid; HSDH, hydroxysteroid dehydrogenases; isoUDCA, isoursodeoxycholic acid; LCA, lithocholic acid; TC/HDLc, ratio of total cholesterol to high-density lipoprotein cholesterol; UCA, ursocholic acid; UDCA, ursodeoxycholic acid; the original names of other BAs are detailed in Table S3.

Although animal studies have delineated the dominant role of gut microbes in producing SBAs and modulating the BA pool, data in free-living human populations are still lacking. To address this gap, we leveraged gut metagenomic data collected alongside fecal BA data to elucidate the relationship between the gut microbiome and fecal BAs, with a focus on SBAs. We first examined the association between the overall structure of the gut microbiome and fecal BAs. Using the first principal coordinate (PCo1) to summarize the overall composition of both the SBA and PBA pools, we observed a stronger association between the PCo1 of the SBA pool and the gut microbial community structure (Spearman correlation coefficient= −0.55) compared to the association for the PBA pool (coefficient = 0.23). Additionally, the PCo1s of the SBA and PBA pools were significantly correlated (coefficient= −0.50). We also found a significant association between the PCo1 of SBA and the gut microbial community structure, as determined by permutational multivariate analysis of variance (PERMANOVA, 999 permutations, R^2^ = 2.6%, *p* = 0.001, Figure 4a). In addition, we categorized participants into four groups based on the median of PBAs and SBAs and found that the microbial composition significantly differed across the four groups (PERMANOVA, R^2^ = 2.3%, *p* = 0.001). These findings suggest that the overall microbial community structure is potentially associated with the conversion of PBAs to SBAs. However, we must note that the relationship between SBAs and the gut microbiome is bidirectional, as SBAs can exert selective pressure on microbial populations.^3,9^ At the same time, PBAs are crucial in determining the levels of SBAs in the gut.

Complementary to the community structure analysis, per-feature association analysis was performed based on a curated list of bacteria and microbial enzymes known for their roles in BA metabolism (Methods and Table S9–10) using multi-variable adjusted linear regression models in MaAsLin2.^24^ Notably, *R. gnavus* and *R. torques*, which encode enzymes 3β-HSDH and 7β-HSDH involved in the oxidation and epimerization of BAs,^25^ showed a positive association with several oxidized or epimerized BA forms (Figure 4b and Table S9, *q* < 0.05), such as UCA, UDCA, and isoUDCA. Several *Bacteroides* spp. known for their deconjugation activity^3^ were inversely associated with various conjugated BAs (*q* < 0.05), such as taurodeoxycholic acid (TDCA) and taurolithocholic acid (TLCA).

Beyond the curated list of species, additional taxa known to participate in BA metabolism were significantly linked to fecal BA levels (Fig. S10). *Megamonas funiformis*, *M. hypermegale* and *Fusicatenibacter saccharivorans*, members of the Firmicutes phylum participating in BA epimerization processes,^3^ showed positive associations with UCA, UDCA, and isoUDCA. *Alistipes putredinis* and *A. shahii* (Bacteroidetes phylum) were inversely associated with several conjugated BAs such as TDCA, TLCA, glycoursodeoxycholic acid (GUDCA), and tauroursodeoxycholic acid (TUDCA).

Furthermore, we observed significant associations between enzymes involved in SBA production and their respective BA products. For example, 7β-HSDH, catalyzing the conversion of UDCA from CDCA and UCA from CA,^12^ was positively associated with levels of UDCA and UCA (*q* < 0.001, Figure 4c and Table S11). Similarly, a higher abundance of 3β-HSDH, which is responsible for biotransformations of iso-BA pathways,^9,12^ was associated with elevated fecal isoUDCA levels (*q* < 0.001). Moreover, we observed higher levels of LCA and DCA in individuals with higher abundance of 7α-dehydroxylation (*q* < 0.05), which involves the removal of a hydroxyl group at the C7 position and is responsible for the conversion of DCA from CA and LCA from CDCA.^26^, These findings not only confirm the role of gut microbes in SBA metabolism but also enhance our understanding of how gut microbial communities modulate the secondary BA pool on a population scale.

To further understand the role of gut microbes in the effects of SBAs on cardiometabolic risk, we investigated whether the association of SBAs with cardiometabolic risk differs based on individuals’ gut microbial profiles. Our findings revealed that the associations of fecal SBAs with BMI and serum lipids were modified by the presence or absence of individual microbial species, such as *R. torques and B. longum*. (Figure 4d and Table S12). For example, the associations between 3-HCOA and BMI (*p* for interaction*=*0.010), TDCA and triglycerides (*p* for interaction*=*0.016), as well as HCA and TC/HDLc (*p* for interaction*=*0.021), were more pronounced in non-carriers of *R. torques*. This suggests that *R. torques* may metabolize these BAs into forms with reduced or altered effects on body adiposity and lipid metabolism. Similarly, *B. longum*, a gram-positive bacterium known for its involvement in the hydrolysis or amidation of BAs,^27^, modified the associations between LCA and BMI (*p* for interaction = 0.008), 7,12-diketo-lithocholic acid (7,12diketo-LCA) and triglycerides (*p* for interaction*=*0.048), as well as isoUDCA and TC/HDLc (*p* for interaction < 0.001). The association of fecal BA levels with BMI and lipid profiles also varied by carriers/non-carriers of several other BA metabolizers, including *Eggerthella lenta, R. gnavus, and B. bifidum* (Fig. S11, *p* for interaction < 0.05). Results from subgroup analyses of the potential interactions between baseline SBAs and gut microbial species were consistent across subgroups stratified by baseline levels of MedDiet adherence index, BMI, triglycerides, and TC/HDL-C ratio, as well as in different age groups and in male participants only (as 88.4% of participants were male), showing similar directional effects on the interaction between BAs and microbial species on body adiposity and lipid biomarkers with the primary analysis (Fig. S12).

## Discussion

Over the past few decades, our understanding of the physiological effects of BAs and the role of gut microbes in their metabolism, particularly regarding lipid and glucose metabolism and immune functions, has advanced significantly. However, much of the current evidence stems from *in vivo* or *in vitro* studies or focuses on circulating BA profiles in humans. There remains a limited understanding of the BA pool in the human gut and its interactions with the host diet and commensal microbial communities. To address these gaps, we conducted a multi-omics analysis within a rigorously executed RCT involving well-characterized human participants. We identified fecal BAs, including specific isoforms of LCA, HCA, and UDCA, as novel predictive biomarkers of body adiposity and serum lipid biomarkers. Additionally, we demonstrated that the modification of biomarkers of cardiometabolic health benefits induced by MedDiet interventions varied by the baseline fecal BA profiles. Our findings also corroborated the crucial role of the gut microbiome in regulating the composition of the gut BA pool, a phenomenon previously documented through *in vitro* and animal models, and extended this knowledge by pinpointing specific microbial taxa that modulate the relationship between fecal BAs and cardiometabolic health in humans.

Prior *in vitro* and *in vivo* studies over the past decades demonstrated that BAs act as critical signaling molecules that regulate a wide array of metabolic pathways via interactions with host BA receptors, including the TGR5 and nuclear receptors such as the FXR and pregnane X receptor, and subsequent gut peptide secretion, including GLP-1.^10^ However, prospective human studies examining the association between fecal BAs and cardiometabolic health are limited. Plasma BAs have been linked to adiposity and lean cardiometabolic risk factors,^13,14^ yet investigations employing fecal BAs – a more accurate indicator of secondary BA profiles – are limited. Recently, Xiao *et al*. reported correlations between fecal DCA and UDCA with higher BMI levels.^28^ Panayiotis *et al*.’s^15^ study in Twins UK Study identified cross-sectional associations between fecal DCA, UCA, and isoUDCA, with post-prandial lipid levels (triglycerides) after an acute meal challenge. Taylor *et al*.^29,^ reported cross-sectional associations between certain fecal HCA and UDCA species, including HCA, 12-DHCA, UCA, and UDCA, with serum triglycerides. Our findings further suggested that the relationship between fecal BAs and TC/HDLc is predominantly influenced by HDLc levels. Moreover, the profiles of BAs associated with body adiposity slightly differ from those associated with triglycerides and TC/HDLc. This distinction may reflect BAs’ dual role: their hormonal effects on various receptors affecting multiple metabolic pathways such as insulin resistance and energy balance, and their direct influence on host lipid metabolism beyond these hormonal interactions.^30^

The MedDiet, characterized by its high consumption of diverse plant foods (including nuts, vegetables, fruits, legumes, and cereals), low intake of red meat and refined grains, preference for olive oil as the main source of fat, fresh fruits for dessert, regular consumption of fish and seafood, limited dairy products, and low-to-moderate wine consumption, has been causally linked to a reduced risk of cardiovascular diseases and type 2 diabetes.^23,31,32^ Our study presents new evidence demonstrating that fecal SBAs, such as TLCA-S and GCOA-S, modulated MedDiet’s effects on body adiposity. Our findings are supported by multiple lines of evidence indicating that BAs are essential for emulsifying dietary fats and facilitating the absorption of lipids and lipophilic vitamins via the formation of mixed micelles.^10^ Furthermore, animal studies have shown that BAs can influence dietary energy harvest^33,34^ and appetite by altering gut microbiome composition and activating TGR5 signaling.^35^ The greater BMI improvement following MedDiet interventions in individuals with higher levels of BA-sulfation isoforms can be attributed to sulfation’s unique properties: enhanced water solubility, diminished intestinal absorption, and increased fecal excretion, that collectively aid in mitigating cholestasis and promoting fat excretion.^36^ Moreover, BAs facilitate dynamic communication between the host and microbiota, serving as a distinctive class of metabolites implicated across the entire spectrum of cardiometabolic disease progression and potentially influencing individual responses to dietary interventions targeting metabolic health.^8,37^

Our findings corroborate and extend those of earlier, predominantly mechanistic studies by linking specific enzymes and bacteria known to possess hydroxysteroid dehydrogenase activity – critical in the oxidation and epimerization of BAs – with epimerized isoforms of fecal BAs^38,39^ in human populations. Furthermore, our novel findings demonstrate the role of microbial metabolizers of BAs in modulating the relationship between fecal BAs and cardiometabolic biomarkers. This observation aligns with findings from animal studies, suggesting that gut microorganisms extend their influence beyond BA biotransformation and are directly involved in the activation of BA signaling pathways, subsequently impacting metabolic risk factors.^40^ These findings open avenues for further research into the systemic effects of gut microbiome-BA interactions on host metabolism, which holds promise for developing strategies such as fecal microbiota transplantation and the application of probiotics/synbiotics for weight management.^41^ Detailed mechanistic studies are needed to explore how specific microbial metabolites, such as short-chain fatty acids or other byproducts of BA metabolism, may modulate the activation of key receptors like TGR5 and FXR, thereby impacting lipid metabolism pathways. Investigating these interactions at the molecular level could elucidate the dynamic regulatory mechanisms through which the gut microbiome influences metabolic health. This knowledge could inform the development of targeted interventions, such as the use of probiotics or dietary modifications, to optimize gut microbiome function, enhance BA metabolism, and mitigate cardiometabolic risk through personalized nutrition strategies.

Our study possesses several strengths. Firstly, the basis of our investigation is a rigorously conducted RCT, which not only ensures the causal integrity of dietary interventions but also benefits from a prospective design. Secondly, the integration of multiple types of molecular measures, including LC-MS for BA quantification and shotgun metagenomics for microbial profiling, offers complementary insights into the mechanisms underlying how gut microbial BA metabolizers influence the BA pool and, subsequently, cardiometabolic risk. However, our study is limited by the predominance of male participants and the inclusion of participants at high metabolic risk, which may limit the generalizability of our findings. Additionally, despite careful adjustments for a wide range of potential confounders, we cannot rule out the possibility of residual confounding and the association between fecal BAs and cardiometabolic biomarkers could also be influenced by other unmeasured factors. Nonetheless, the convergence of data from various assays and the consistency of these results with prior *in vivo* and *in vitro* research lend substantial support to the causality of our observations and significantly diminish the likelihood that our conclusions can be attributed to residual confounding factors.

## Conclusion

In summary, we identified fecal BAs as novel biomarkers for predicting body adiposity and lipid profiles, as well as individuals’ differential responses to MedDiet-style dietary interventions. Furthermore, we have elucidated the intricate role of the gut microbiome in the complex interactions among dietary intake, BA metabolism, and metabolic health outcomes. This work advances the field of precision nutrition and opens a promising avenue for the development of tailored dietary strategies aimed at enhancing cardiometabolic health. The dynamic interplay between gut microbiome diversity and BA signaling pathways offers therapeutic potential for reducing the risk of cardiometabolic diseases. Future studies are clearly needed to investigate the mechanistic pathways that underpin the diet-BA interaction and its influence on metabolic health, thereby paving the way for innovative approaches to more effective prevention and management of metabolic disease.

## Methods

### Study population

The DIRECT-PLUS trial (clinicaltrials.gov ID: NCT03020186) was designed to evaluate the effects of Mediterranean diet versus standard healthy dietary guidelines (HDG, control group) on body adiposity and serum biomarkers of cardiometabolic risk. Conducted from May 2017 to November 2018 in an isolated workplace (Nuclear Research Center Negev, Dimona, Israel), this intervention trial provided participants with monitored lunches. Clinical measurements, biospecimen collection, and lifestyle intervention sessions were administered in the medical department of the workplace. The trial included 294 men and women over 30 years old who had either abdominal obesity (waist circumference: men >102 cm, women >88 cm) or dyslipidemia (Triglycerides >150 mg/dL and high-density lipoprotein cholesterol [HDLc] ≤40 mg/dL for men, ≤50 mg/dL for women) at enrollment. Our prior publications have reported significant reductions in body adiposity and improvements in serum cardiometabolic biomarkers with MedDiet interventions compared to the control group.^11^ Exclusion criteria are detailed in Supplemental Methods 1. Participants were allocated in a 1:1:1 ratio to HDG, MedDiet, and Green-MedDiet. The MedDiet arm was instructed to adhere to a calorie-restricted Mediterranean dietary pattern, consistent with protocols in our previous studies.^11,19,42^ The MedDiet prioritized a high consumption of vegetables, substituted poultry and fish for beef and lamb, and included a daily intake of 28 g of walnuts. The Green-MedDiet group received the standard MedDiet and daily walnut provision with additional enhancements, including 3–4 cups/day of green tea and 100 g/day of *Wolffia globosa* (*Mankai* strain) in the form of frozen cubes, served as a green shake.^43^ All intervention groups received the same format and intensity of physical activity interventions, which included free gym memberships and educational sessions to engage in moderate-intensity physical activity (Table S2). This study collected fecal and blood samples at baseline, 6, and 18 months during the intervention. Detailed descriptions of the intervention protocols and study execution are available in Supplemental Methods 2–4 and Table S2. The study was approved by the Soroka University Medical Centre Institutional Review Board. Participants provided written informed consent and received no compensation. The present analysis included 284 participants who donated fecal and blood samples at baseline.

### Measurement of bile acids in fecal samples

Fecal samples were collected at the study site, immediately frozen at −20°C for 1–3 days, and subsequently stored at −80°C until laboratory assays.^11,19^ Metabolon, Inc. (Durham, NC, USA) performed BA profiling using ultrahigh-performance liquid chromatography with tandem mass spectrometry (UPLC-MS). Briefly, the fecal samples were prepared, analyzed, and processed using standard protocols.^44^ All samples were randomized across assay runs with pooled reference samples spaced evenly among the injections as quality control. The lab personnel was blinded to intervention assignments and sample collection times. BAs were identified by comparing sample features with ion features in a reference database of in-house synthesized standard compounds, followed by detailed visual inspection and quality control. Raw data from a total of 44 BAs were extracted, and their fecal levels were quantified as the area under the peak. This study included 39 distinct BA isoforms that were above the detection threshold in more than 20% of the samples, as well as the sum of PBAs, SBAs, and the ratio of PBAs to SBAs. We imputed missing values using half of the minimum detectable fecal level for each specific BA.

### Metagenomic sequencing of fecal samples

The processing of fecal samples and the subsequent shotgun metagenomic sequencing were conducted at the Alkek Center for Metagenomics and Microbiome Research at Baylor College of Medicine, Houston, TX, USA. DNA was extracted using the Qiagen DNeasy PowerSoil Pro Kit. Libraries for sequencing were prepared using Illumina DNA Prep, with each sample uniquely identified by a barcode using kit-appropriate Unique Dual Index adapter sets. Quality control involved PicoGreen (Thermo), Qubit (Invitrogen), Fragment Analyzer (Agilent), and Tapestation (Agilent) for concentration and fragment size evaluation. The pooled libraries underwent shotgun sequencing on the Illumina NovaSeq platform, following a 2 × 150bp paired-end protocol. Raw fastq sequences were demultiplexed and processed using BBDuk, which included quality trimming, Illumina adapters removal, and PhiX reads filtering. These trimmed FASTQ files were then aligned to a combined PhiX (standard Illumina spike in), and host reference genome database using BBMap to eliminate host/PhiX reads.

### Microbiome taxonomic and functional profiling

We generated taxonomic and functional profiles of the gut microbiome using the bioBakery 3 meta’omics workflow.^45^ Sequencing reads underwent quality control using the KneadData 0.7.0 pipeline with default parameters to remove low-quality read bases and human-originating reads. Taxonomic profiling was conducted with MetaPhlAn 3.0,^46^ which classifies metagenomic reads to taxonomies and outputs relative abundances of identified taxa. Functional profiling was performed using HUMAnN 3.0.0.^46^ Reads were recruited to sample-specific pangenomes using Bowtie2.^47^ Unmapped reads were aligned against UniRef90^48^ by applying DIAMOND translated search.^49^ The resulting UniRef90 abundances were then mapped to level 4 Enzyme Commission (EC) nomenclature.^50^ Leveraging the established evidence regarding the microbial metabolism of BAs,^3,9,12,39^ our downstream analysis of the associations between the microbial features and fecal BA levels mainly focused on 14 candidate microbial species within the genera *Blautia*, *Bifidobacterium, Clostridium, Bacteroides, Eggerthella*, and *Escherichia*, and enzymes, including bile salt hydrolases (BSH), 7α-dehydroxylase, and HSDH (Supplemental Methods 5). Furthermore, we conducted hypothesis-free analyses as a complementary secondary approach.

### Dietary assessment and measurement of other covariables

The DIRECT-PLUS Trial employed a validated food frequency questionnaire (FFQ)^51,52^ to assess dietary intake, wherein participants self-reported dietary intake at baseline, 6, and 18 months during the intervention. We calculated the average daily intake of nutrients and foods based on the reported frequency and portion sizes of each food item from the FFQ. The adherence to the MedDiet was assessed using a MedDiet index based on the Mediterranean diet pyramid.^53,54^ This index summarized intake levels across eight foods and nutrients: vegetables, legumes, fruits, nuts, whole grains, red/processed meats, fish, and alcohol. For beneficial components (vegetables, legumes, fruit, nuts, whole grains, fish), individuals with consumption below the median were assigned a value of 0, while those with consumption at or above the median were assigned a value of 1. For red/processed meat intake, participants with consumption below the median were assigned a value of 1, and those with consumption at or above the median were assigned a value of 0. For alcohol consumption, men consuming between 10 and 25 g/day and women consuming between 5 and 15 g/day were assigned a value of 1, while those outside these ranges were assigned a value of 0. Additionally, lifestyle information, such as physical activity levels, was collected via a computer-based questionnaire, and medication information was extracted from self-reported records. Physical activity intensity was measured in metabolic equivalent of task (MET) units per week, where each unit represents the ratio of work metabolic rate to resting metabolic rate.^55^

### Measurement of body adiposity and serum biomarkers

The primary outcomes of our study included body adiposity, as quantified by body mass index (BMI), and serum biomarkers of lipid metabolism. Study personnel performed physical examinations and collected biospecimens at baseline, month 6, and month 18. Height and weight were measured using a standard stadiometer and precision scale. BMI was calculated as weight in kilograms divided by height in meters squared. Blood samples, collected at 8:00 am after a 12-hour fasting period, were centrifugated and stored at −80°C. Serum concentrations of triglycerides, total cholesterol (TC), HDLc, and low-density lipoprotein cholesterol (LDLc) were determined enzymatically using a Cobas-6000 automatic analyzer (Roche) at the University of Leipzig, Germany.

## Statistical analysis

We applied the inverse normal transformation to BA levels to achieve approximately Gaussian distributions and stabilize the variance. To evaluate the prospective associations between baseline BA levels and the outcomes longitudinally measured at three-time points, taking into consideration the design involving repeated measures, we employed generalized estimating equation (GEE) models to perform individual tests for each BA species in the association and interaction analyses, which effectively handle repeated measures data while accounting for within-subject correlations. The GEE models in association evaluation quantified the multivariable-adjusted mean difference in body adiposity or lipid biomarker levels, with their 95% confidence intervals (CIs), associated with one standard deviation increase in baseline BA levels. The models included body adiposity or lipid biomarkers as dependent variables, individual BA species as independent variables, and age, sex, time, antibiotic use, metformin use, lipid-lowering medication use, MedDiet index, and intervention assignment as covariates. Participant identifiers were used to specify the correlation structure, accounting for within-subject correlations. In the GEE model, each response variable $Y_{\mathrm{ij}}$ for the ith subject at the jth measurement is associated with a p× *1* vector of covariates $X_{\mathrm{ij}}$, which includes both time-invariant factors (e.g., sex) and time-varying factors (e.g., time since baseline). The form is:

$$\mu_{\mathrm{ij}}=E\left(Y_{\mathrm{ij}}|X_{\mathrm{ij}}\right)=\beta_{1}+\beta_{2}X_{2\mathrm{ij}}+\ldots +\beta_{p}X_{\mathrm{pij}}$$

The variance of $Y_{\mathrm{ij}}$ is specified to depend on the mean response $\mu_{\mathrm{ij}}$ through: *Var*$\;\left(Y_{\mathrm{ij}}|X_{\mathrm{ij}}\right)=\phi \nu (\mu_{\mathrm{ij}})$. The correlation between repeated measurements for the same subject is modeled through a specified correlation structure. The covariance between responses $Y_{\mathrm{ij}}$ and $Y_{\mathrm{ik}}$ is assumed to be: $\mathrm{Corr}(Y_{\mathrm{ij},}Y_{\mathrm{ik}})=\alpha \;|k-j|(0\leq \;\alpha \;\leq 1)$.

To investigate the potential interaction between MedDiet’s effects and baseline BAs, the GEE model further included an interaction term between baseline BAs and dietary intervention groups alongside terms for their independent effects. The formula is:

$$Y_{\mathrm{ij}}=\beta_{1}+\beta_{2}\mathrm{MedDie}t_{i}+\beta_{3}\mathrm{Bil}e_{i}+\beta_{4}\mathrm{MedDie}t_{i}\times \mathrm{Bil}e_{i}+\ldots +\beta_{p}X_{\mathrm{pij}}$$

A significant testing (false discovery rate adjusted *p-*value <0.05) of the interaction term from the Wald test can be interpreted as a significant interaction between diet and BAs, referred to as a modification. For this analysis, baseline BA levels were dichotomized at the median of each BA. In addition, we used GEE models to assess the impact of dietary interventions on fecal BA levels after 6 and 18 months of intervention. This model specifically incorporated an interaction term between dietary intervention group assignment and the time variable (representing the slope of changes in BA levels over time), in addition to adjusting for covariates and specifying participant identifiers to address within-subject correlations:

$$\mathrm{Bil}e_{\mathrm{ij}}=\beta_{1}+\beta_{2}\mathrm{Meddie}t_{i}+\beta_{3}\mathrm{Tim}e_{\mathrm{ij}}+\beta_{4}\mathrm{MedDie}t_{i}\times \mathrm{Tim}e_{\mathrm{ij}}+\ldots +\beta_{p}X_{\mathrm{pij}}$$

We then tested the significance of the beta coefficient of the interaction term using a two-sided likelihood ratio test by comparing models with and without the interaction term to determine the *p*-value for interaction.

We further investigated the role of gut microbiome in BA metabolism and its potential modulation of the associations of fecal BAs with BMI and lipid biomarkers through targeted analysis of candidate microbial features and data-driven, hypothesis-free analysis. For the latter, microbial species and functions were required to have a minimum relative abundance of 0.001% in at least 10% of samples. Post-filtering, 151 microbial species, 1570 level-4 ECs, and 372 biochemical pathways met these criteria. We employed permutational multivariate analysis of variance (PERMANOVA, 999 permutations) based on Bray-Curtis dissimilarity calculated from species-level taxonomic data to assess the association between SBAs and microbial community structure. We performed principal coordinate analysis (PCoA, based on the Euclidean distance) to summarize BA levels, with the first principal coordinate (PCo1) used as a summary metric of the measured BA pool in the gut. Furthermore, we categorized participants into four groups based on median values of PBAs and SBAs and assessed their association with microbial community structure using PERMANOVA. We used total sum scaling to generate the relative abundance of microbial features and log-transformed the relative abundance before including them in the association and interaction analysis. Associations between SBAs and microbial species and functions were examined through linear regression analysis in MaAsLin2,^24^ adjusting for the covariables including age, sex, antibiotic use, metformin use, and lipid-lowering medication use. To determine if specific microbial species modified the associations of BA levels with BMI and lipid biomarkers, we included interaction terms between BA levels and microbial species carriage status in the aforementioned GEE models with simultaneous adjustment for the covariates, and specifying participant identifiers to handle within-subject correlations. All microbial species were dichotomized based on carrier/noncarrier at baseline for this analysis. *P*-values from the analyses were adjusted for multiple comparisons using the Benjamini-Hochberg method to control the false discovery rate (FDR) at a target of 0.05 for *q*-values. All statistical analyses were conducted using R version 4.2.3.

## Supplementary Material

Supplemental Material

## Data Availability

The majority of results corresponding to the current study are included in the article or uploaded as supplementary materials. Due to the informed consent of the participants, for access to the individual-level deidentified data from the DIRECT-PLUS Study, please direct your request to Drs. Iris Shai and Dong D. Wang.

## Code availability statement

This study mainly relies on open-source bioinformatic tools described in detail in Methods. The analysis-specific programs are available through https://github.com/DW-Group/BileAcids_DIRECT-PLUS.
