# Supplementary material for: Gut microbial metabolism of bile acids modifies the effect of Mediterranean diet interventions on cardiometabolic risk in a randomized controlled trial
Source: Gut Microbes. 2024 Nov 13;16(1):2426610. doi: 10.1080/19490976.2024.2426610 (PMC11567240; doi:10.1080/19490976.2024.2426610)
Supplement: Supplemental Material [file KGMI_A_2426610_SM3405.zip › KGMI 2426610/Supplemental Methods and Figures.docx]

Supplemental Methods and Figures

Supplemental Methods

Supplemental Methods 1: Exclusion criteria

Exclusion criteria included: inability to engage in physical activity, a serum creatinine level of no less than 2 mg/dL, disturbed liver function, a major illness with potential hospitalization requirements, pregnancy or lactation for women, presence of active cancer or undergoing chemotherapy either at present or in the prior three years, participation in another trial, chronic treatment with warfarin (due to its potential interaction with vitamin K), and the presence of a pacemaker or platinum implant (may hinder participation in the magnetic resonance imaging included in the study design).

Supplemental Methods 2: Physical activity protocol

The aerobic exercise regimen began with 20 minutes of training at 65% of maximum heart rate, progressively increasing to 45-60 minutes at 80% of maximum heart rate. The full workout program encompassed 45-60 minutes of aerobic training 3-4 times a week. The resistance training component started with a single set using weights at 60% of the maximum weight, eventually progressing to two sets at 80% of the maximum. The resistance training included a variety of exercises including leg extensions, leg curls, squats, lateral pull-downs, push-ups, shoulder presses, elbow flexions, triceps extensions, and bent leg sit-ups.

Supplemental Methods 3: Lifestyle sessions and motivation techniques

The lifestyle interventions consisted of 90-minute sessions focusing on nutrition and physical activity, conducted in the workplace and facilitated by a multidisciplinary team including physicians, clinical dietitians, and fitness instructors. These sessions were held weekly during the first month, and then once a month for the subsequent interventions. All three participant groups received these educational programs at the same level of intensity. To keep the participants motivated, text messages with relevant information for each assigned intervention group were sent at fixed time intervals. Furthermore, a dedicated website providing all necessary nutritional and physical activity information needed by the participants to continue with the intervention was accessible to the participants according to their intervention group.

Supplemental Methods 4: Plant and Polyphenol-rich foods, provided at no cost to participants

Walnuts (groups MedDiet, Green-MedDiet): The main polyphenols in walnuts are ellagitannins, ellagic acid, and their derivatives. The nutrition composition of walnuts (28g), taken from the USDA: energy:183 kcal, protein: 4.3g, lipids: 18.3g, carbohydrates: 3.8, fiber: 1.9g, total saturated fatty acids-1.7g, total monounsaturated fatty acids-2.5g, total polyunsaturated fatty acids-13.2g.

Green tea (group Green-MedDiet): an unfermented tea produced from the leaves of Camellia sinensis, prepared by drying and steaming the leaves, and is a rich source of polyphenols. Most of the polyphenols found in green tea are catechins (the monomer form of flavanols), mainly epigallocatechin (EGC), epicatechin gallate (ECG), and epigallocatechin gallate (EGCG). The participants were required to pour heated water over the green tea bag and steep for 3 minutes.

*Wolffia globosa* duckweed-*Mankai* (group Green-MedDiet): A specific strain of *Wolffia globosa*, an aquatic plant in the duckweed family, can serve as a plant protein source. Nutritionally, *Mankai* is characterized by high protein content (more than 45% of the dry matter) and the presence of 9 essential and 6 conditional amino acids. In addition, it is a good source of omega-3 fatty acids. The *Mankai* plant is rich in insoluble fibers, vitamins, minerals (including iron and zinc), and polyphenols. The nutrition composition of *Mankai* (values for 100gr frozen cubes *Mankai* shake (~20g dry): Energy, 80 kcal; protein, 9g; lipids, 1.6g; carbohydrates, 7.5; fiber, 1.9g; total saturated fatty acids,1.7g. *Mankai* provides bioavailable essential amino acids and iron and has a beneficial effect on postprandial and fasting glycemic control. We guided the participants to prepare a green *Mankai* shake with additional ingredients, which were also part of the diet regimen (fruits, walnuts, or vegetables) each evening. The green protein shake was partially substituted for dinner, replacing beef/poultry protein sources.

Supplemental Methods 5: Microbiome statistical analysis

Microbiome composition was assessed based on relative abundance. We log-transformed relative abundances of microbial features before including them in the association or interaction analysis. We focused on 14 candidate microbial species that have been previously reported participating in secondary BAs metabolism (*Ruminococcus* spp., *Clostridium* spp., *Bifidobacterium* spp., *Bacteroides* spp., *Eggerthella lenta, and Escherichia coli*; Table below).^1-3^ We examined the association between these microbial species and fecal BA levels through multi-variable adjusted linear regression analysis. In addition, we examined whether the association of BAs with cardiometabolic health markers can be modulated by the presence/absence of specific species. Several transformations of BAs by the gut microbiota^1,2,4,5^ are summarized below**：**

| **Microbial producers** | **Genus** | **BA transformation examples** | **Enzymes catalyzing reactions** | **Modification** |
| --- | --- | --- | --- | --- |
| *Eggerthella lenta* | *Eggerthella* | DCA→3-oxoDCA  3-oxoDCA→isoDCA  CDCA→7-oxoLCA  DCA→12-oxoLCA  CDCA→UDCA  CA→7,12-dioxoLCA | 3α-hydroxysteroid  dehydrogenases (HSDH)  3β-HSDH  7α-HSDH  12α-HSDH | Oxidation  Epimerization |
| *Escherichia coli* | *Escherichia* | CDCA→7-oxoLCA | 7α-HSDH | Oxidation |
| *Ruminococcus gnavus*  *Ruminococcus torques* | *Blautia* | DCA→3-oxoDCA  3-oxoDCA→isoDCA  7-oxoLCA→UDCA  CDCA→UDCA  CA→3-oxoCA | 3α-HSDH  3β-HSDH  7β-HSDH | Oxidation, Epimerization |
| *Clostridium spp.* | *Clostridium* | CDCA→LCA  CA→DCA  DCA→12-oxoLCA | BA-inducible (Bai) operon  12α-HSDH  Bile salt hydrolases (BSH) | Dehydroxylation  Desulfation  Hydrolysis |
| *Bifidobacterium spp.*  *Bacteroides spp.* | *Bifidobacterium*  *Bacteroides* | DCA→oxoLCA  Conjugated  →unconjugated BAs  Unconjugated →conjugated BAs | BSH | Amidation  Hydrolysis |

References:

1. Cai J, Sun L, Gonzalez FJ. Gut microbiota-derived bile acids in intestinal immunity, inflammation, and tumorigenesis. *Cell host & microbe*. 2022; 30:289-300. doi: 10.1016/j.chom.2022.02.004. PMID: 35271802.

2. Collins SL, Stine JG, Bisanz JE, Okafor CD, Patterson AD. Bile acids and the gut microbiota: metabolic interactions and impacts on disease. *Nat Rev Microbiol*. 2023; 21:236-247. doi: 10.1038/s41579-022-00805-x. PMID: 36253479.

3. Krautkramer KA, Fan J, Bäckhed F. Gut microbial metabolites as multi-kingdom intermediates. *Nature reviews Microbiology*. 2021; 19:77-94. doi: 10.1038/s41579-020-0438-4. PMID: 32968241.

4. Doden HL, Wolf PG, Gaskins HR, Anantharaman K, Alves JMP, Ridlon JM. Completion of the gut microbial epi-bile acid pathway. *Gut Microbes*. 2021; 13:1-20. doi: 10.1080/19490976.2021.1907271. PMID: 33938389.

5. Lucas LN, Barrett K, Kerby RL, Zhang Q, Cattaneo LE, Stevenson D, Rey FE, Amador-Noguez D. Dominant Bacterial Phyla from the Human Gut Show Widespread Ability To Transform and Conjugate Bile Acids. *mSystems*. 2021:e0080521. doi: 10.1128/mSystems.00805-21. PMID: 34463573.

**Supplemental Figures**


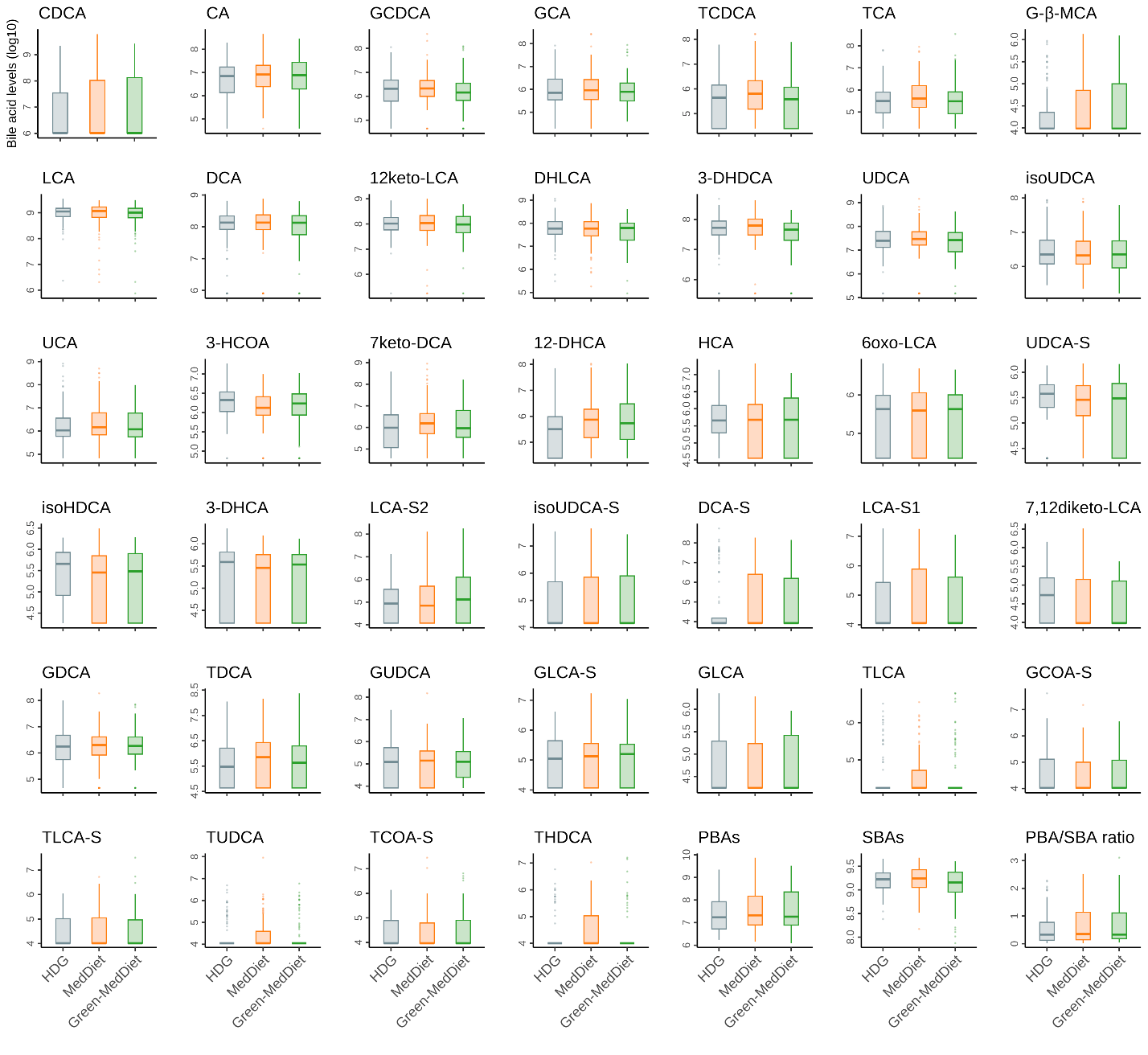


**Figure S1.** Distributions of baseline fecal bile acid levels across three dietary intervention groups. The center of each box plot was the median value, with the boxes displaying the interquartile ranges, and upper and lower whiskers indicating 1.5 times the interquartile range from above the upper quartile and below the lower quartile, respectively. The original names of bile acids are detailed in Table S3.


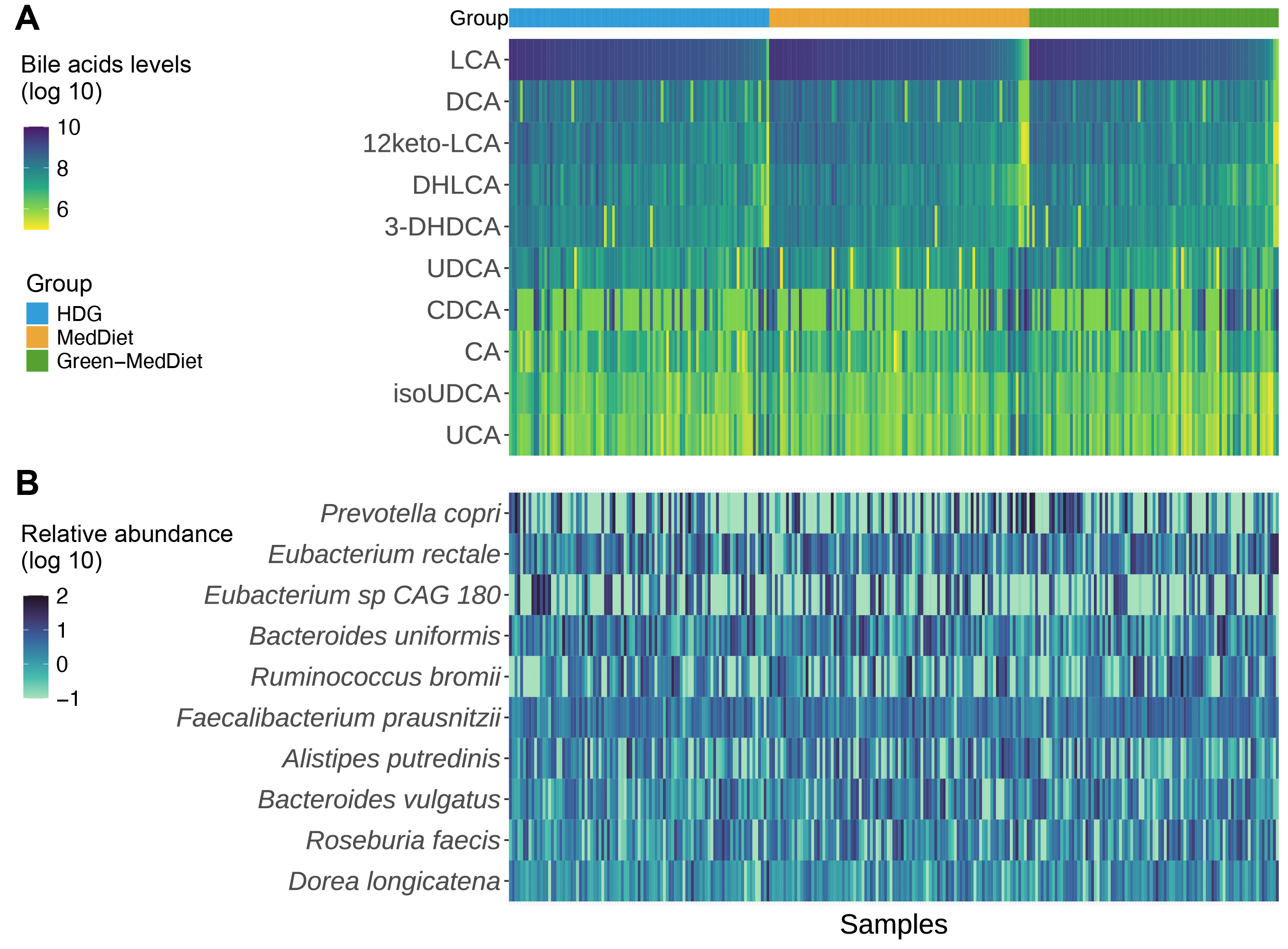


**Figure S2.** The distribution of fecal bile acids and gut microbiome taxonomy profiles. **(A)** Distributions of the 10 bile acids most abundant on average in analyzed fecal bile acids. **(B)** Distributions of the 10 microbial species most abundant on average in the gut microbiome. Abbreviations: CA, cholic acid; CDCA, chenodeoxycholic acid; DCA, deoxycholic acid; DHLCA, dehydrolithocholic acid; isoUDCA, isoursodeoxycholic acid; LCA, lithocholic acid; UCA, ursocholic acid; UDCA, ursodeoxycholic acid; 3DHDCA, 3-dehydrodeoxycholic acid; 12keto-LCA, 12-ketolithocholic acid.


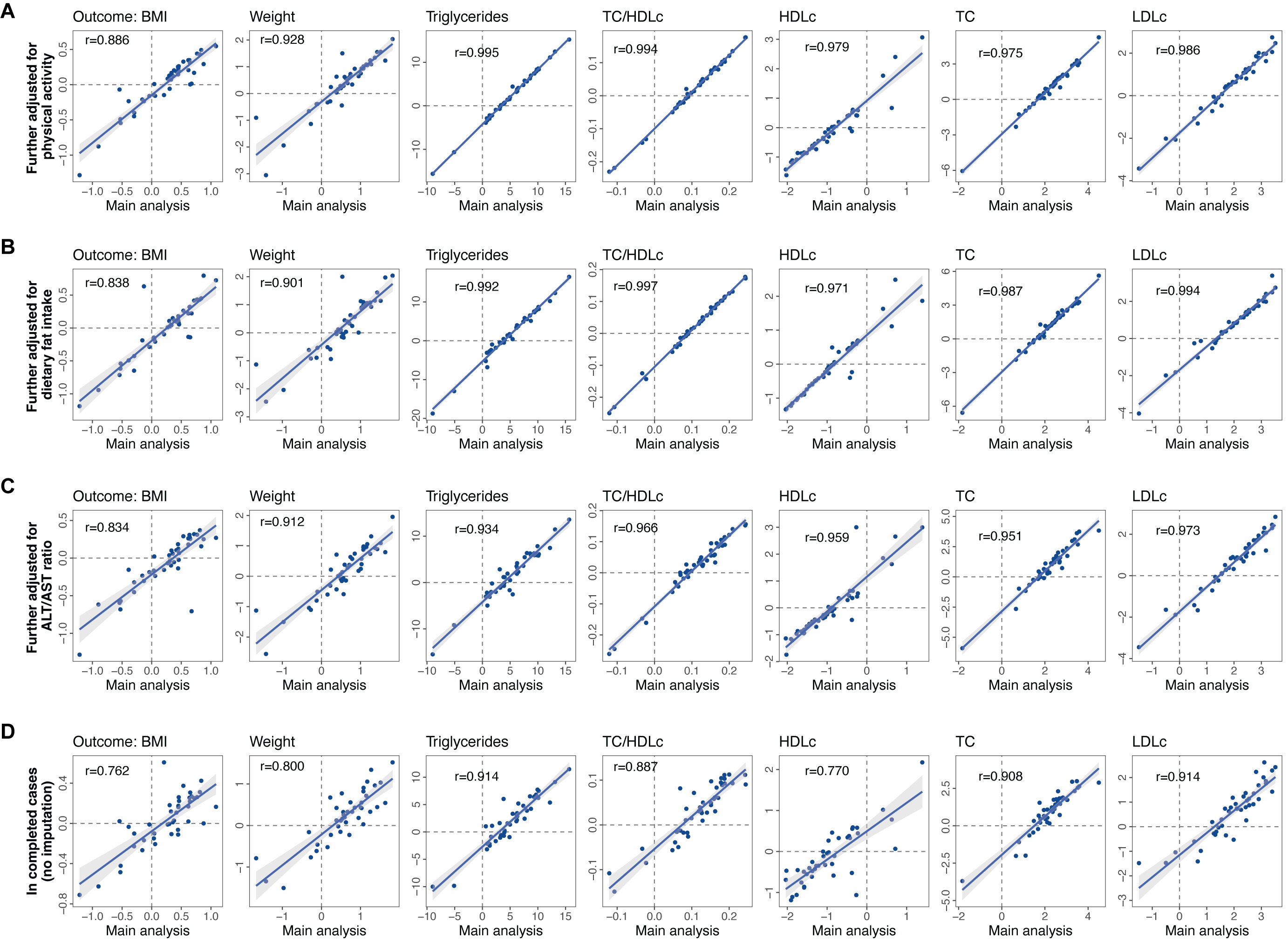


**Figure S3.** Comparisons in associations between baseline bile acids (BAs) and cardiometabolic health biomarkers in the main analysis and the others that further adjusted for **(A)** physical activity, **(B)** dietary fat intake, **(C)** alanine aminotransferase/aspartate aminotransferase (ALT/AST) ratio, and those that **(D)** removed samples with missing values for BAs that had a missing rate between 10% and 80%. Dots in the scatter plots represent beta coefficients for the BA-cardiometabolic health biomarker associations from the multivariable-adjusted GEE models. Spearman correlation coefficients for these beta coefficient values were calculated.


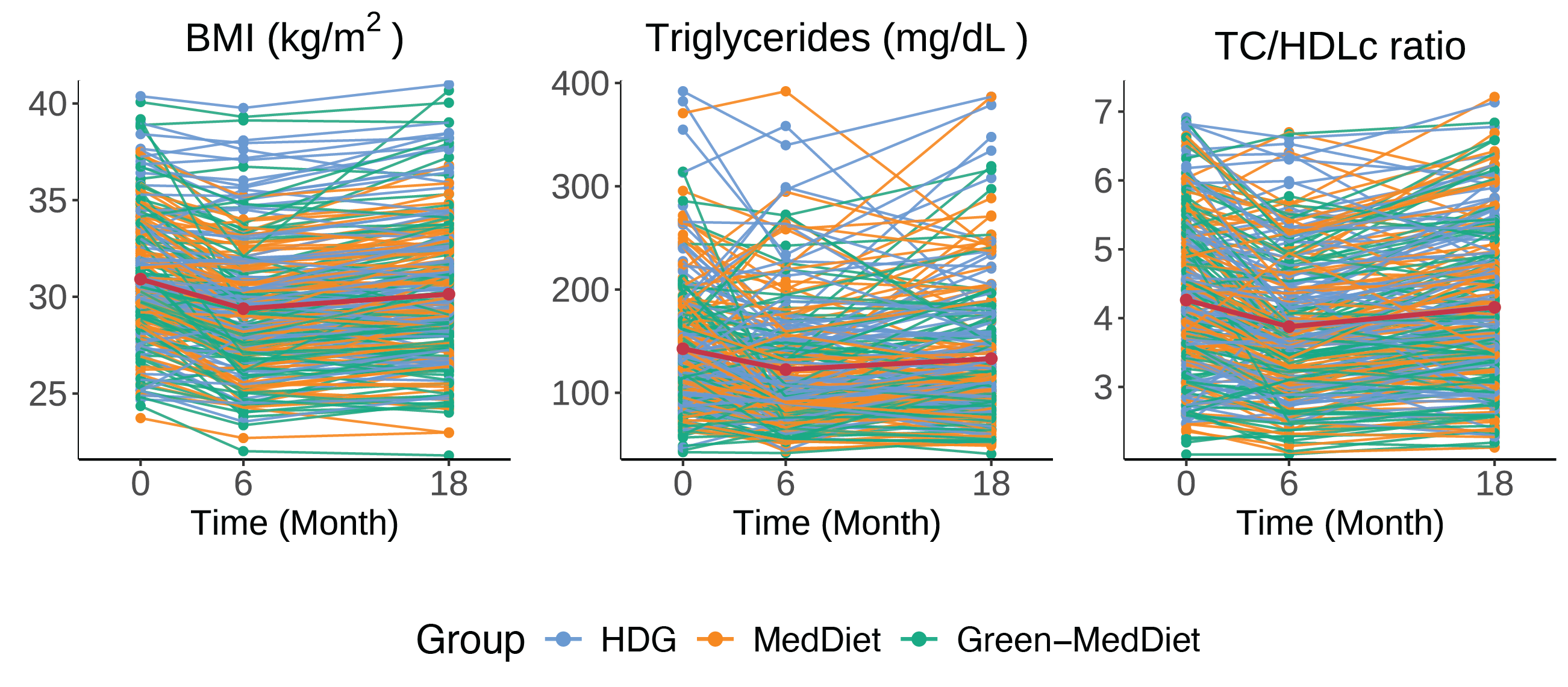


**Figure S4.** Temporal trends in body mass index (BMI) and lipid biomarkers among individuals in different dietary intervention groups. The red points with connected lines represent the average values of the outcome indicators at each time point and the trend over time. Abbreviations: HDG, healthy dietary guidelines; MedDiet, Mediterranean diet; TC/HDLc, the ratio of total cholesterol to high-density lipoprotein cholesterol.


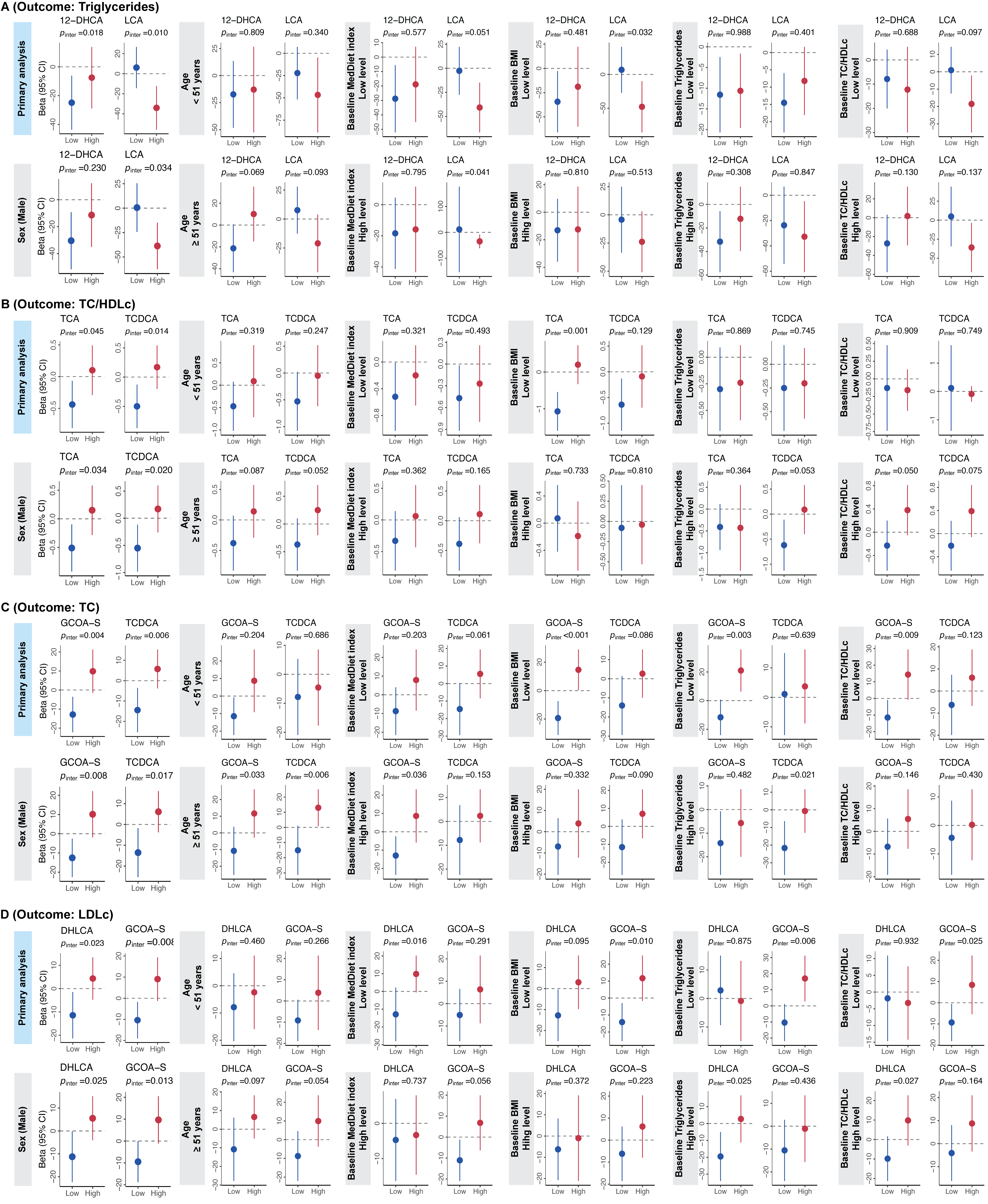


**Figure S5.** The interaction between baseline bile acid (BA) levels and Mediterranean diet (MedDiet) interventions on lipid biomarkers including **(A)** triglycerides, **(B)** total cholesterol to high-density lipoprotein cholesterol (TC/HDLc) ratio, **(C)** TC, **(D)** LDLc, was analyzed in the primary analysis. This interaction was also examined in subgroups, including male participants and subgroups based on age, and baseline levels of MedDiet adherence index, BMI, triglycerides, and TC/HDL-C ratio. Low and high levels were defined by the median values of the corresponding group indicator. The dots in the plot represent the beta coefficients for the effect of the two MedDiet groups combined compared to the healthy dietary guideline (HDG) group on lipid biomarkers in different BA level groups from multivariable-adjusted generalized estimating equation (GEE) models, with whiskers indicating the upper and lower limits of the 95% confidence intervals (CIs). Low vs. high BA levels were determined based on the median values of each BA. We calculated the *p*-values for the interaction terms between intervention group assignment and BAs using the multivariable-adjusted GEE models. Abbreviations: 12-DHCA, 12-dehydrocholic acid; DHLCA, dehydrolithocholic acid; GCOA-S, glycocholenoic acid sulfate; LCA, lithocholic acid; TCA, taurocholic acid; TCDCA, taurochenodeoxycholic acid.


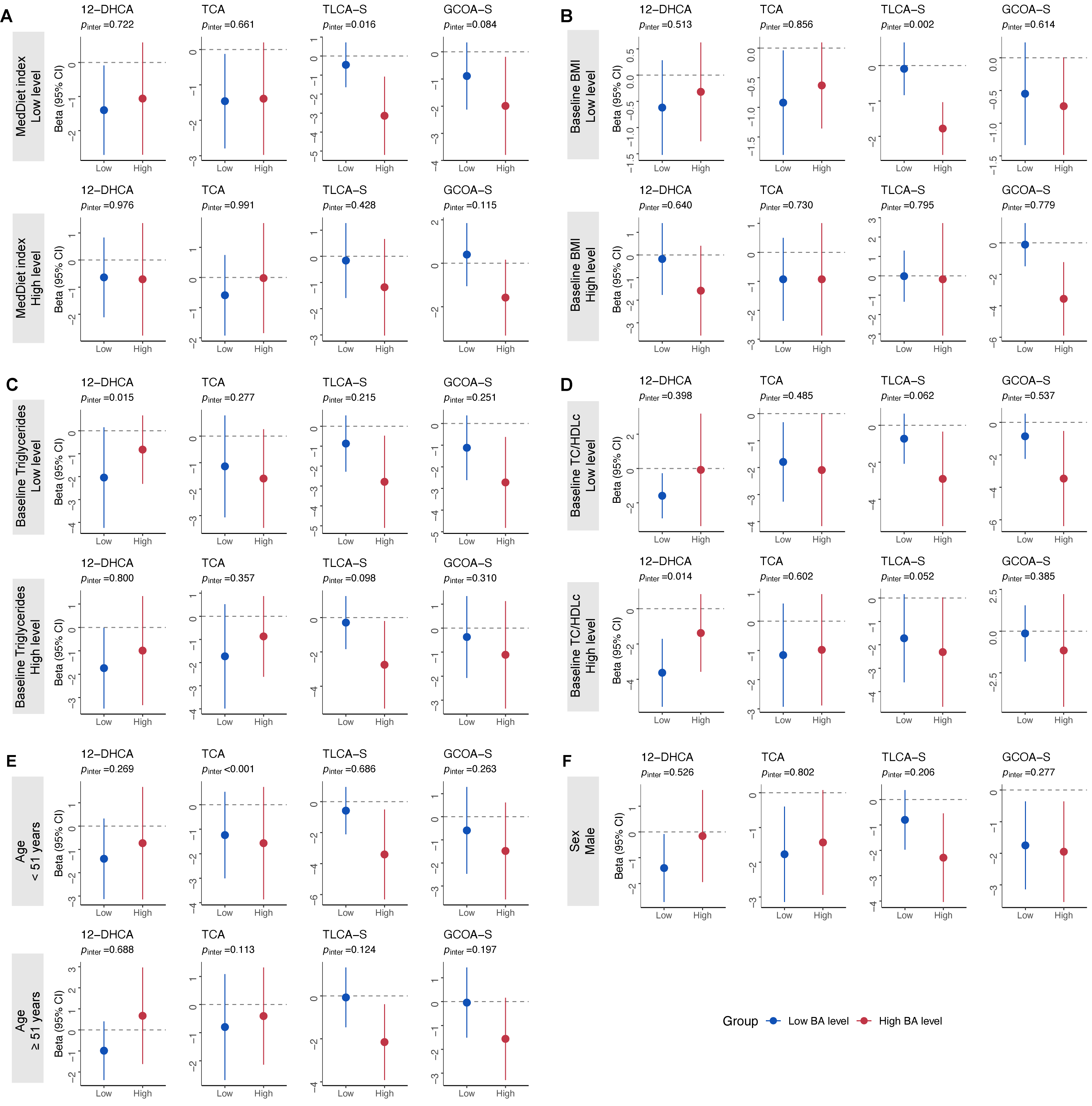


**Figure S6.** The interaction between baseline bile acid (BA) levels and Mediterranean diet (MedDiet) interventions on body mass index (BMI) in different subgroups based on **(A)** baseline MedDiet adherence index, **(B)** baseline BMI, **(C)** baseline triglycerides, **(D)** baseline total cholesterol to high-density lipoprotein cholesterol [TC/HDLc] ratio, **(E)** age, as well as in **(F)** male participants. Low and high levels were defined by the median values of the corresponding group indicator. The dots in the plot represent the beta coefficients for the effect of the two MedDiet groups combined compared to the healthy dietary guideline (HDG) group on BMI in different BA level groups from multivariable-adjusted generalized estimating equation (GEE) models, with whiskers indicating the upper and lower limits of the 95% confidence intervals (CIs). Low vs. high BA levels were determined based on the median values of each BA. We calculated the *p*-values for the interaction terms between intervention group assignment and BAs using the multivariable-adjusted GEE models. Abbreviations: GCOA-S, glycocholenoic acid sulfate; TCA, taurocholic acid; TLCA-S, taurolithocholic acid 3-sulfate; 12-DHCA, 12-dehydrocholic acid.


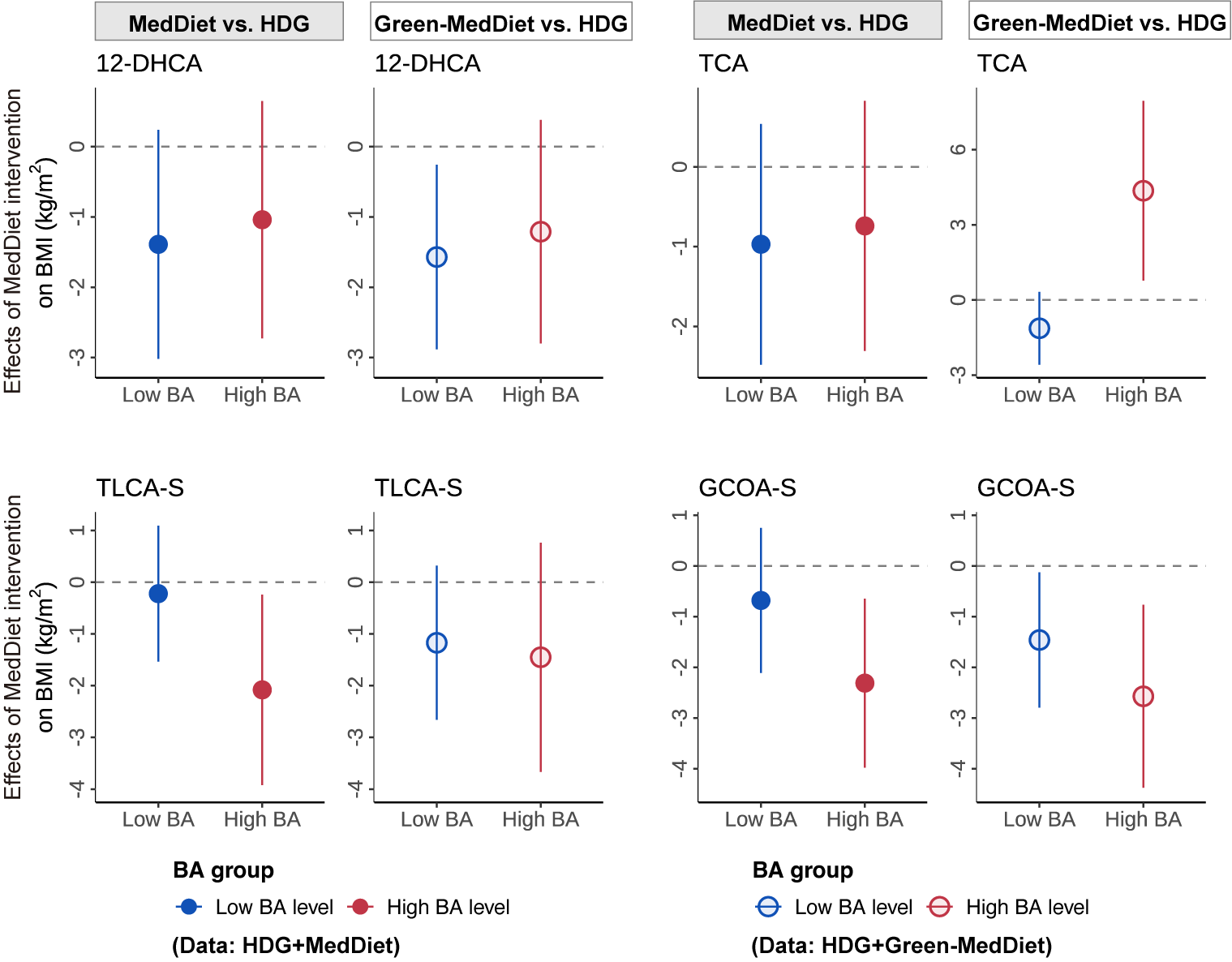


**Figure S7**. Effects of dietary intervention on body mass index in participants with low vs. high baseline level of bile acid (BA) for Mediterranean diet (MedDiet) and Green-MedDiet separately. The dots in the plot represent the beta coefficients for the effect of the MedDiet arm vs. healthy dietary guideline (HDG) group, or Green-MedDiet arm vs. HDG, on BMI in different BA level groups from multivariable-adjusted generalized estimating equation (GEE) models, with whiskers indicating the upper and lower limits of the 95% confidence intervals (CIs). Low vs. high BA levels were determined based on the median values of each BA. We calculated the p-values for the interaction terms between intervention group assignment and BAs using the multivariable-adjusted GEE models. Abbreviations: GCOA-S, glycocholenoic acid sulfate; TCA, taurocholic acid; TLCA-S, taurolithocholic acid 3-sulfate; 12-DHCA, 12-dehydrocholic acid.


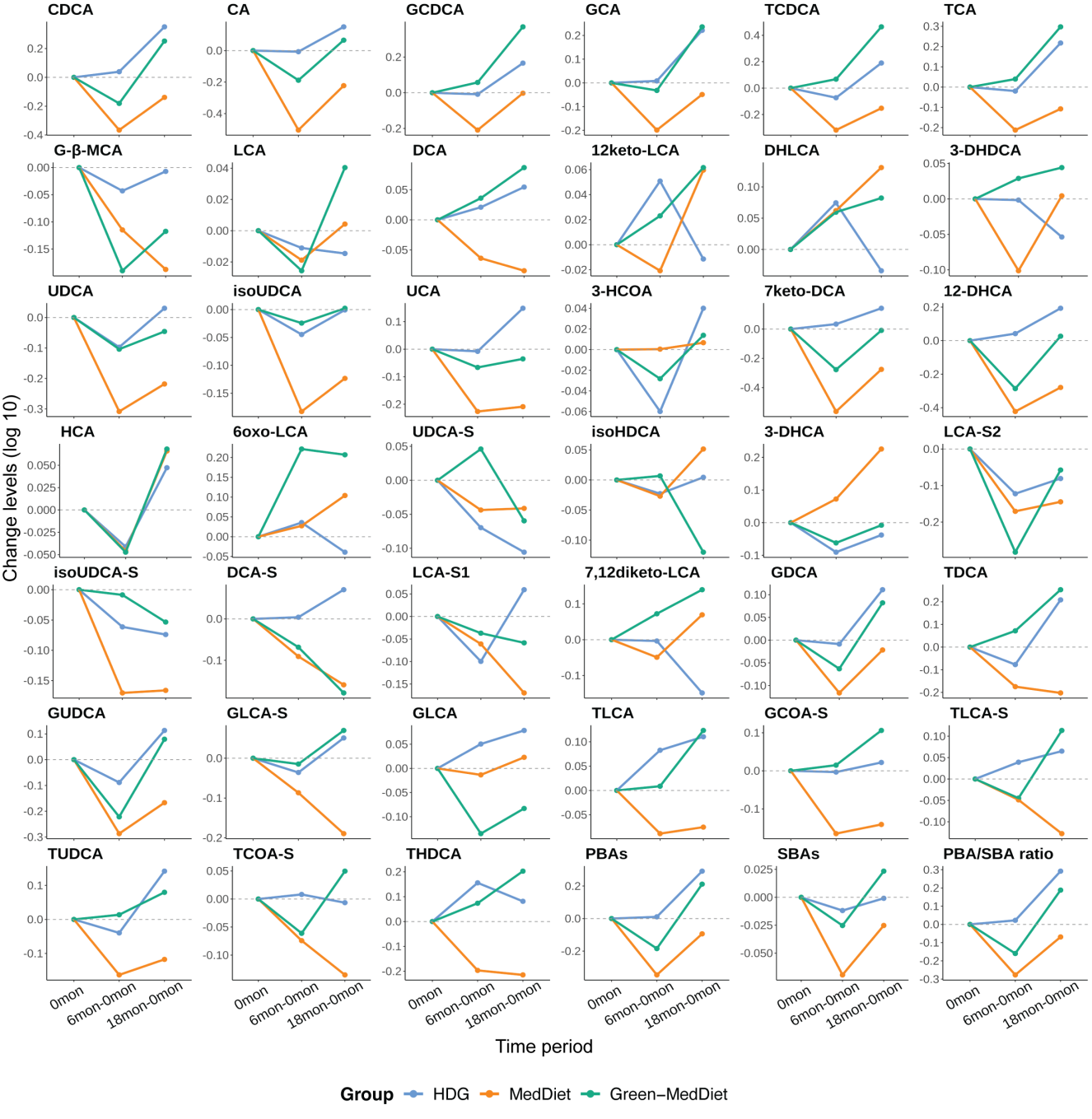


**Figure S8.** The distribution of change levels in fecal bile acids over different time periods in three dietary intervention groups. Points represent the average change levels of each bile acid at baseline, month 6 versus baseline, and month 18 versus baseline. The original names of bile acids are detailed in Table S3.


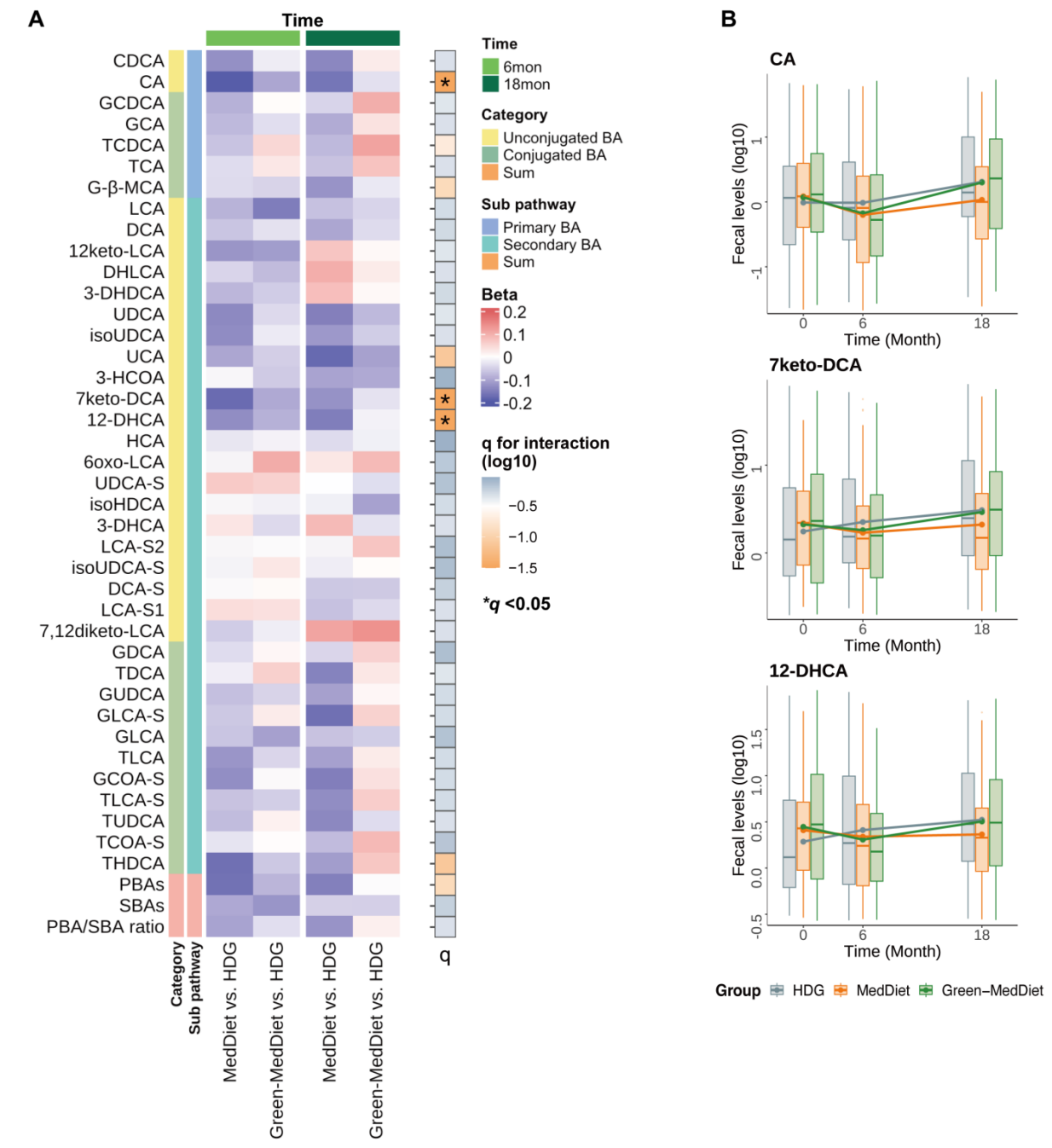


**Figure S9.** The effect of Mediterranean diet on fecal bile acid levels after 6 and 18 months of intervention. (A) Beta coefficients were derived from multivariable-adjusted GEE models, by comparing the Mediterranean diet (MedDiet) and Green-MedDiet groups to the healthy dietary guidelines (HDG) group, respectively. The significance level of the interaction between dietary intervention groups and time was derived from the likelihood ratio test in the multivariable-adjusted generalized estimating equation models. (B) Temporal trends in fecal bile acid levels in three intervention groups. Abbreviations: CA, cholic acid; 7keto-DCA, 7keto-deoxycholic acid; 12-DHCA, 12-dehydrocholic acid.
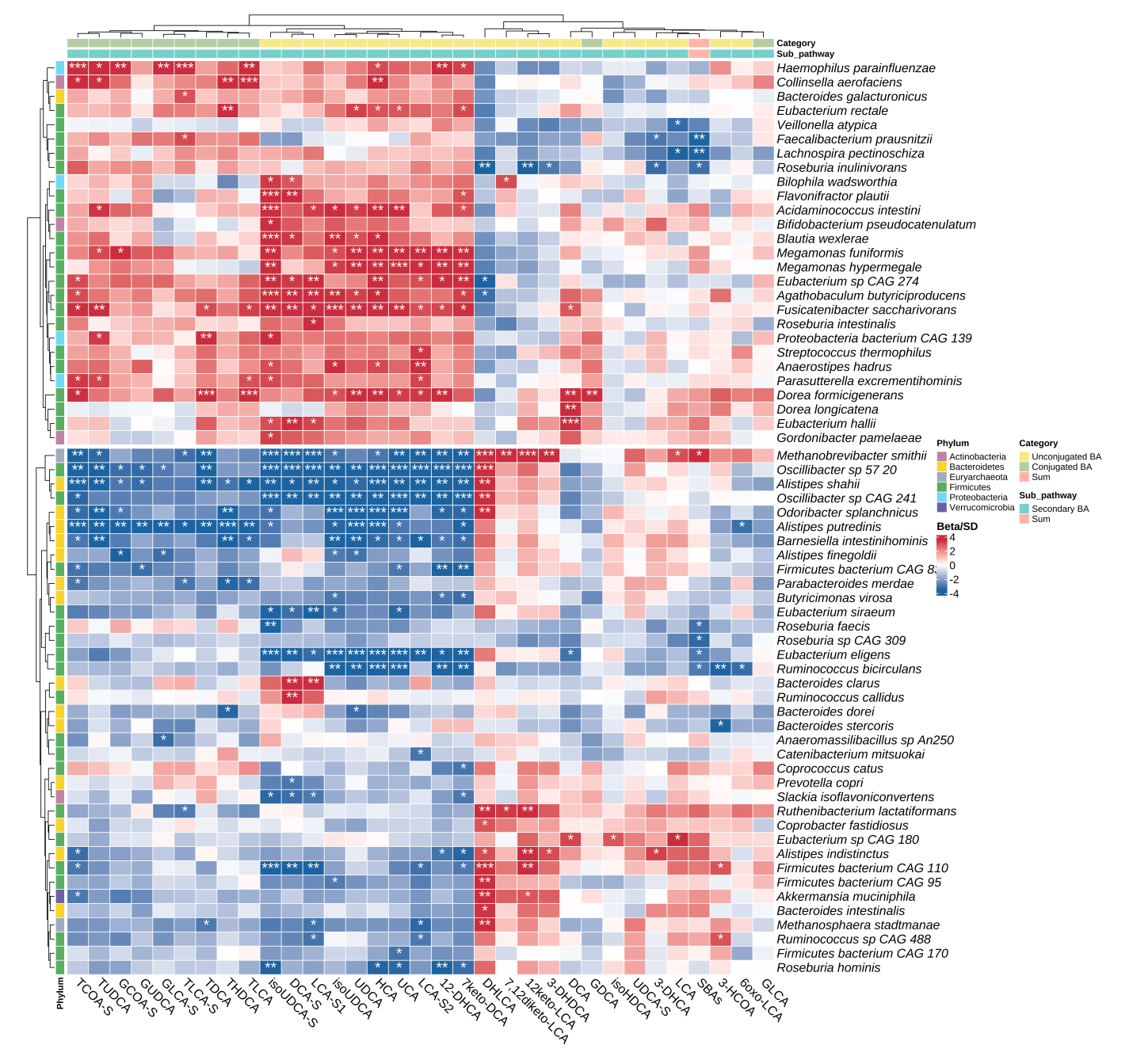


**Figure S10.** Associations of secondary fecal bile acid levels at baseline with species-level features. The colors of the heatmap are in correspondence to the values of beta coefficient/standard deviation from linear regression models simultaneously adjusted for age, sex, antibiotic use, metformin use, and lipid-lowering medication use. Statistical significance is from the linear model with multiple comparison adjustments using the Benjamini-Hochberg method to calculate *q*-values (false discovery rate adjusted *p*-value). The original names of bile acids are detailed in Table S3. All the statistical tests were two-sided. * 0.01≤ *q* <0.05, ** 0.001≤ *q* <0.01, *** *q* <0.001.


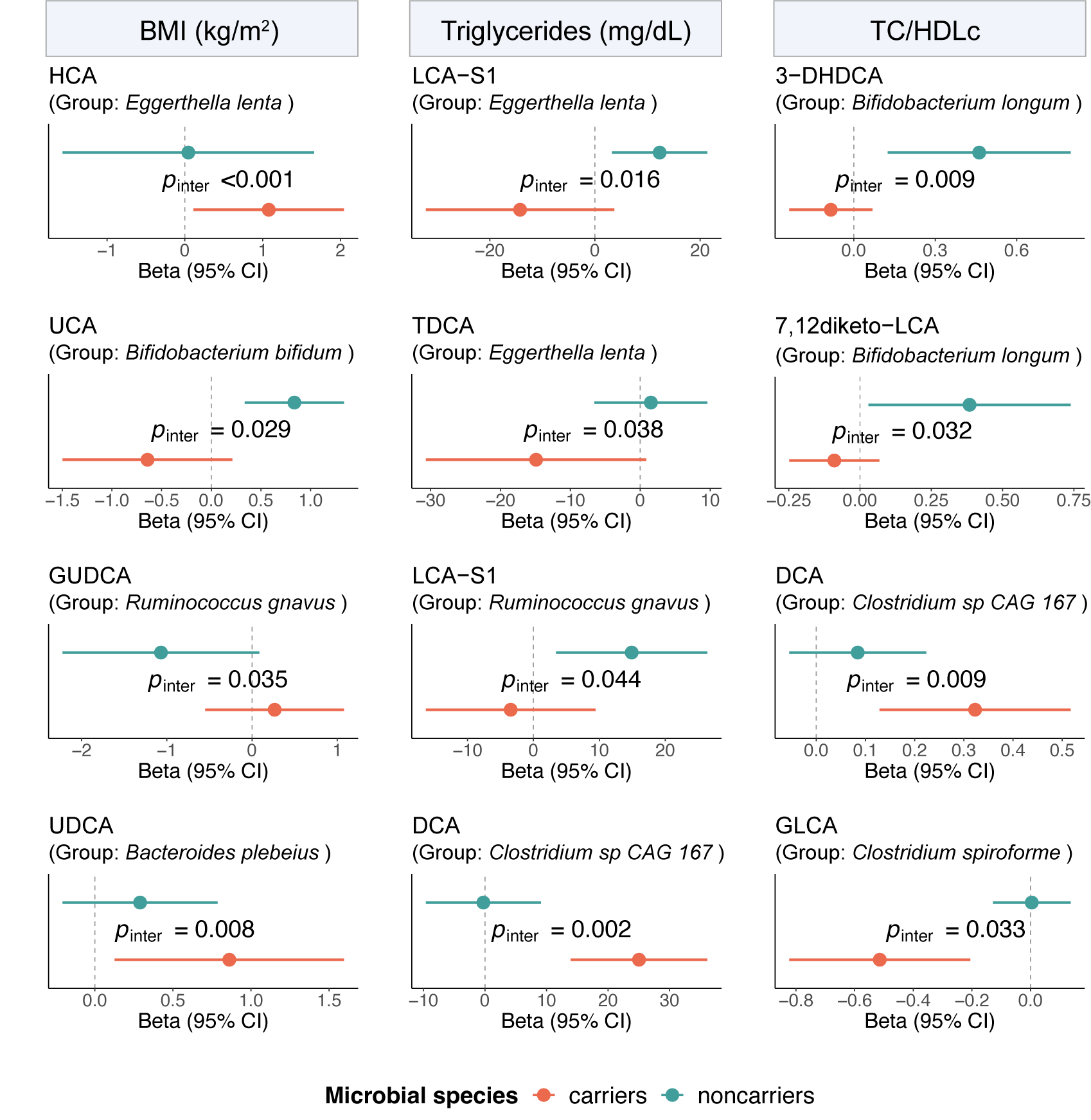


**Figure S11.** The presence or absence of certain microbes modified the association of fecal secondary bile acids (BAs) with body adiposity and lipid biomarkers. The dot plots with whiskers indicate the difference in body mass index (BMI) per one-standard deviation increment in BA levels with 95% confidence intervals (CIs) in carriers and noncarriers of microbial species. The original names of bile acids are detailed in Table S3.


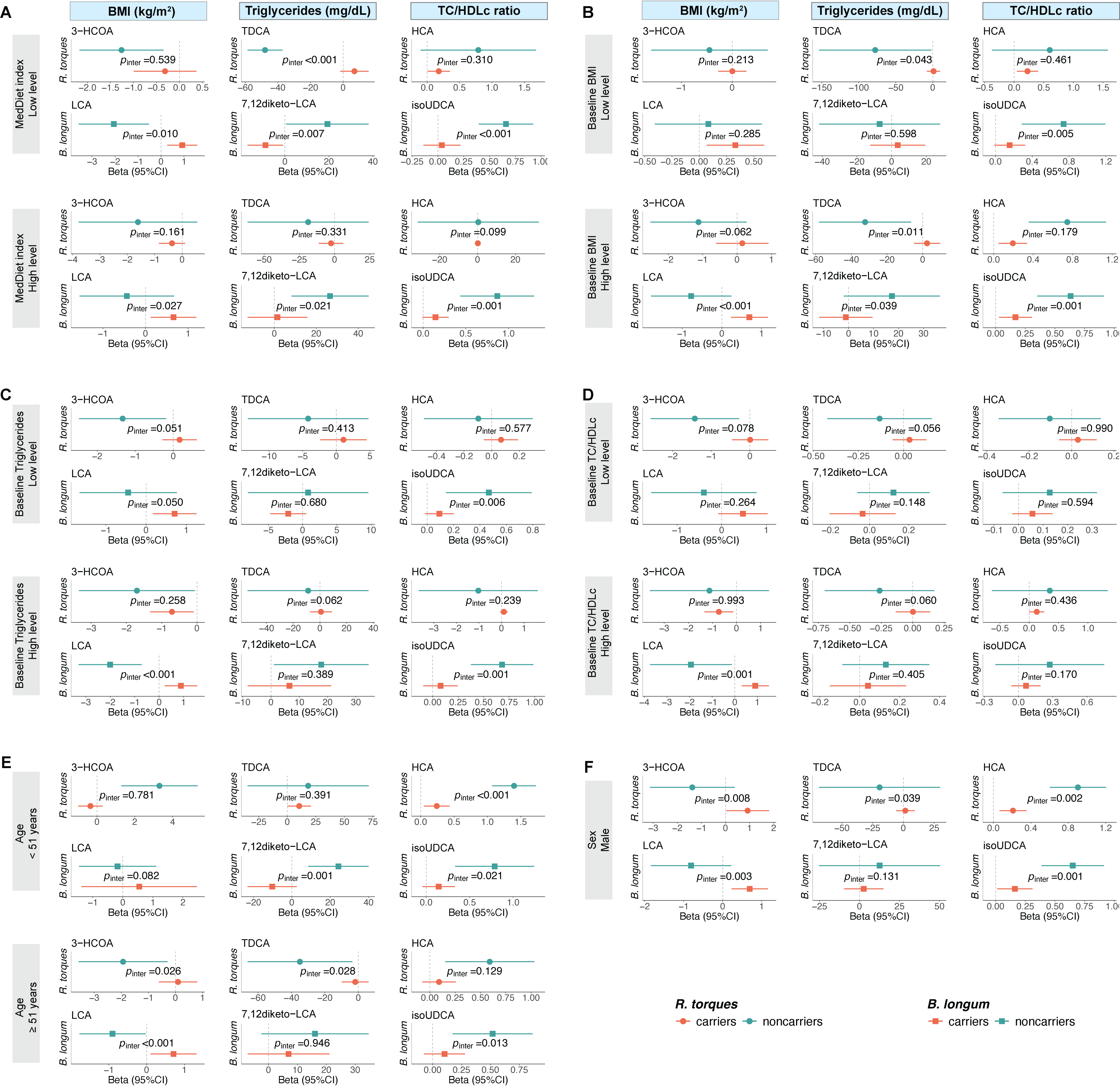


**Figure S12.** The association of fecal secondary bile acids with body adiposity and serum lipid biomarkers stratified by gut microbial species across different groups based on **(A)** baseline Mediterranean diet adherence index, **(B)** baseline body mass index (BMI), **(C)** baseline triglycerides, **(D)** baseline total cholesterol to high-density lipoprotein cholesterol (TC/HDLc) ratio, **(E)** age, as well as in **(F)** male participants were almost consistent with the main results. The dot plots with whiskers indicate the difference in outcome per one-standard deviation increment in bile acid (BA) levels with 95% confidence intervals (CIs) in carriers and noncarriers of the specific species. Low and high levels were defined by the median values of the corresponding group indicator. The original name of each BA are detailed in Table S3.
